# Progression Risk Assessment of Post-surgical Papillary Thyroid Carcinoma Based on Circular RNA-Associated Competing Endogenous RNA Mechanisms

**DOI:** 10.3389/fcell.2020.606327

**Published:** 2021-01-21

**Authors:** Mengwei Wu, Shuo Li, Jiashu Han, Rui Liu, Hongwei Yuan, Xiequn Xu, Xiaobin Li, Ziwen Liu

**Affiliations:** ^1^Department of General Surgery, Peking Union Medical College Hospital, Chinese Academy of Medical Sciences & Peking Union Medical College, Beijing, China; ^2^MD Program, Chinese Academy of Medical Sciences & Peking Union Medical College, Beijing, China

**Keywords:** nomogram, papillary thyroid carcinoma, ceRNA, circRNA, progression-free interval

## Abstract

**Background:** Accurate risk assessment of post-surgical progression in papillary thyroid carcinoma (PTC) patients is critical. Exploring key differentially expressed mRNAs (DE-mRNAs) regulated by differentially expressed circular RNAs (circRNAs) via the ceRNA mechanism could help establish a novel assessment tool.

**Methods:** ceRNA network was established based on differentially expressed RNAs and correlation analysis. DE-mRNAs within the ceRNA network associated with progression-free interval (PFI) of PTC were identified to construct a prognostic ceRNA regulatory subnetwork. least absolute shrinkage and selection operator (LASSO)–Cox regression was applied to identify hub DE-mRNAs and establish a novel DE-mRNA signature in predicting PFI of PTC.

**Results:** Six hub DE-mRNAs, namely, *CLCNKB, FXBO27, FXYD6, RIMS2, SPC24*, and *CDKN2A*, were identified to be most significantly related to the PFI of PTC, and a prognostic DE-mRNA signature was proposed. A nomogram incorporating the DE-mRNA signature and clinical parameters was established to improve the progression risk assessment in post-surgical PTC, which was superior to the American Thyroid Association risk stratification system and distant Metastasis, patient Age, Completeness of resection, local Invasion, and tumor Size (MACIS) score American Joint Committee on Cancer staging system.

**Conclusions:** Based on the circRNA-associated ceRNA RNA mechanism, a DE-mRNA signature and prognostic nomogram was established, which may improve the progression risk assessment in post-surgical PTC.

## Introduction

Thyroid cancer, originating from thyroid follicular epithelial cells, is the most common malignancy of the endocrine system (La Vecchia et al., 2015). Thyroid cancer is currently the eighth most common malignancy around the world (Antonelli and La Motta, 2017), and its incidence is rapidly increasing worldwide. There are four main types of thyroid cancer, including papillary thyroid carcinoma (PTC), follicular thyroid carcinoma, anaplastic thyroid carcinoma, and medullary thyroid carcinoma. Of these, PTC accounts for more than 80% of all thyroid cancer cases. Among all types of thyroid cancer, PTC has the highest incidence with a higher proportion of young patients. Genetic mutations and environmental exposures are the major risk factors for PTC in which *BRAF* V600E is the most common mutation. The primary treatment for PTC is thyroidectomy. Most PTCs grow slowly, and 90% of patients have a relatively good prognosis after treatment, with a low post-operative recurrence rate <10% (Haugen et al., 2016). However, a small percentage of patients experience a high risk of recurrence and metastasis after surgery, resulting in a poor prognosis.

The molecular mechanisms leading to PTC recurrence remain unclear. Moreover, there is a lack of satisfactory assessment tools to precisely evaluate the post-surgical risk of recurrence in PTC. The American Thyroid Association (ATA) currently recommends the use of American Joint Committee on Cancer (AJCC) staging system and distant Metastasis, patient Age, Completeness of resection, local Invasion, and tumor Size (MACIS) score to predict mortality after PTC and proposed the risk stratification system for risk assessment of post-surgical progression in PTC (Haugen et al., 2016). However, the accuracy of currently available assessment tools is inadequate to meet the requirements of clinical work. The challenge of treating PTC lies in balancing side effects and treatment benefits (Cabanillas et al., 2016). Patients with low- and high-risk PTC should be differentiated to adopt different treatment strategies. Patients with high risk of recurrence require more extensive surgical resection and adjuvant radioactive iodine therapy to improve post-surgical prognosis and prevent recurrence. Secondary surgery for recurrence can result in additional surgical trauma with a higher risk of recurrent laryngeal nerve injury. Alternatively, PTC patients with low risk of recurrence should receive a more conservative treatment to reduce surgical trauma and improve quality of life. Unnecessary post-surgical radioactive iodine therapy and thyroid-stimulating hormone (TSH) suppression therapy should also be avoided. Long-term subclinical hyperthyroidism caused by a high dose of TSH suppression can lead to multiple potential side effects, including osteoporosis, atrial fibrillation, heart discomfort, and an increased risk of fractures and heart disease in elderly patients (Biondi and Cooper, 2018). Unnecessary radioactive iodine therapy also increases the risk of developing other cancers. Therefore, more accurate assessment tools to assess the risk of progression in post-surgical PTC patients are urgently required.

Non-coding RNAs such as microRNAs (miRNAs), long non-coding RNAs, and circular RNAs (circRNAs) are major components of the human transcriptome. circRNAs were first discovered in human cells in 1986 and are critical in the pathophysiology of several human diseases including cancer (Kos et al., 1986). circRNAs are products of a reverse splicing mechanism and are resistant to RNase R (Chen and Yang, 2015). circRNAs contain a single-stranded covalent closed-loop structure that have neither a 5′-3′ polarity nor a polyadenylated tail. circRNAs are primarily located in the cytoplasm and regulate gene expression by acting as an miRNA sponge at the transcriptional or post-transcriptional stage, affecting protein translation and thus the regulation of cellular processes. According to the competitive endogenous RNA (ceRNA) hypothesis, non-coding RNAs such as circRNAs can positively regulate mRNA expression by competitive binding of its shared miRNAs (Salmena et al., 2011). Recent studies further reveal that several circRNAs differentially expressed in cancers can exert a tumor-promoting or tumor-suppressing role via ceRNA mechanism, thus regulating apoptosis, proliferation, invasion, and metastasis (Zhong et al., 2018; Liu et al., 2020). mRNAs regulated by differentially expressed circRNAs (DE-circRNAs) may be more likely to play a functional role in cancer. Particularly in PTC, the upregulated *SOX2* by circ_0005273/hsa-miR-1183 axis promotes the proliferation, invasion, and metastasis of PTC (Zhang et al., 2020). Transforming growth factor α, upregulated by hsa-circ-0060060/hsa-miR-144-3p axis in PTC, was identified to promote the proliferation and autophagy of PTC and was associated with poor prognosis (Liu et al., 2018). The establishment of circRNA–miRNA–mRNA regulatory networks in PTC may provide key targets and novel regulatory pathways in understanding the progression of PTC.

The primary objective of this study was to explore key differentially expressed mRNAs (DE-mRNAs) regulated by DE-circRNA–associated ceRNA regulatory mechanism in PTC to elucidate the post-surgical progression of PTC and establish a novel assessment tool.

## Materials and Methods

### Identification of DE-circRNAs, DE-miRNAs, and DE-mRNAs in PTC

Expression data of circRNAs and related clinical data for PTC were downloaded from the GSE93522 dataset based on GPL19978 platform (Peng et al., 2017). An annotation file provided by the manufacturers was used to match the probes with corresponding circRNA IDs. The median ranking value was used to determine the expression value if multiple probes matched a single circRNA ID. The ‘LIMMA’ R package was used to identify DE-circRNAs between cancer and normal tissues (Ritchie et al., 2015). The cutoff value was set at |Log2FC| > 1 and *P* < 0.05. The basic characteristics of DE-circRNAs were obtained from circBase (https://www.circbase.org/), and the corresponding structures were analyzed using the Cancer-Specific circRNA Database (CSCD) (https://gb.whu.edu.cn/CSCD/#/).

Normalized RNA sequencing data in the form of millions of transcripts (TPM) for miRNA and mRNA and related clinical data of post-surgical PTC were downloaded from The Cancer Genome Atlas (TCGA) (https://portal.gdc.cancer.gov/, up to July 1, 2020). TCGA-Thyroid Cancer (THCA) dataset originally included 507 cases, 510 tumor samples, and 58 normal tissue samples. A total of 495 cases of PTC with matching tumor tissue and clinical information, and 58 normal samples of thyroid tissue were eventually included in the current analysis following the removal of five cases without matching tumor samples, two cases with poorly differentiated oncolytic carcinoma or follicular carcinoma, five cases with history of neoadjuvant therapy, and eight samples of metastases. The ‘LIMMA’ R package was used to identify DE-miRNAs and DE-mRNAs between cancer and normal tissues with a cutoff value set at | Log2FC | > 1 and a false discovery rate (FDR) < 0.05 (Ritchie et al., 2015). GEPIA (http://gepia.cancer-pku.cn) incorporating RNA sequencing expression data of tumors and normal samples from TCGA and the Genotype-Tissue Expression projects was used to validate the differential expression level of a specific DE-mRNA (Tang et al., 2017). Data of copy number alterations and mutation were downloaded from Cbioportal (http://www.cbioportal.org/).

### Construction of the circRNA–miRNA–mRNA Network

CIRCinteractome is a computational tool that enables the prediction and mapping of binding sites for RNA-binding proteins and miRNAs on reported circRNAs (Dudekula et al., 2016). circRNA-miRNA relationships were predicted using CIRCinteractome. miRWalk is an open-source platform that provides predicted and validated miRNA-binding sites with a machine learning algorithm, incorporating data from TargetScan (conserved site context scores, version 7.1), miRDB (release 5.0), and validated information from miRTarBase (version 7.0) (Sticht et al., 2018). miRNA–mRNA relationships were predicted using miRWalk 3.0 with score ≥0.95. Based on differential expression data, only relationship pairs with negative correlation were retained. The correlation between DE-miRNA and DE-mRNA expression with potential ceRNA regulatory relationships was further analyzed using Pearson correlation coefficient in TCGA-THCA dataset. Only miRNA–mRNA relationships with *r* < −0.2 and *P* < 0.05 were retained in the final circRNA–miRNA–mRNA network. To construct a ceRNA regulatory subnetwork associated with progression-free interval (PFI) of PTC, univariate Cox regression analysis was performed based on the DE-mRNAs involved. Only DE-mRNAs associated with PFI of PTC and the corresponding circRNA-miRNA and miRNA–mRNA pairs were retained in the PFI-related subnetwork. The ceRNA network was visualized using Cytoscape v.3.8.0 (https://www.cytoscape.org/). The Sankey diagram that represented the ceRNA regulatory relationship was drafted using Origin 2020 (https://www.originlab.com/).

### Bioinformatics Analysis of the circRNA–miRNA–mRNA Network

To investigate the underlying biological function of ceRNA regulatory relationships in PTC, DAVID (https://david.ncifcrf.gov/) was employed to explore enriched biological processes, cellular components, molecular functions, and significantly relevant signal pathways of DE-mRNAs involved, using default parameters (Huang da et al., 2009). *P* < 0.05 was regarded statistically significant. The results were visualized using the OmicShare tools, a free online platform for data analysis (http://www.omicshare.com/tools).

### Identification of PFI-Related Hub DE-mRNAs and Establishment of the Prognostic DE-mRNA Signature

In this study, PFI was selected as the main endpoint (https://wiki.nci.nih.gov/plugins/servlet/mobile#content/view/24279961). DE-mRNAs associated with PFI were identified based on TCGA-THCA dataset using a univariate Cox proportional hazards regression model. Normalized gene expression data were transformed on the base-2 logarithm for further survival analysis. DE-mRNAs with *P* < 0.05 were considered statistically significant for further analysis. Only cases with follow-up >30 days were included for survival analysis. The 492 eligible TCGA cases were subsequently randomly divided into a training dataset and a validation dataset in a 7:3 ratio. Least absolute shrinkage and selection operator (LASSO) penalized Cox regression analysis was performed to select prognostic hub DE-mRNAs related to PFI and constructed a prognostic DE-mRNA signature in patients with PTC based on a linear combination of the regression coefficient derived from LASSO–Cox regression model coefficients (β) multiplied with its normalized mRNA expression level in the training dataset. Patients were divided into the high-risk and the low-risk group based on the optimal cutoff value determined by X-Tile (Camp et al., 2004). Performance of the prognostic DE-mRNA signature was assessed using Harrell's concordance index (C-index), area under the curve (AUC) of the receiver operating characteristic (ROC) curve, and Kaplan–Meier analysis. The validation dataset and TCGA-THCA dataset were further utilized for validation.

### Gene Set Enrichment Analysis

Gene Set Enrichment Analysis (GSEA) was performed to compare the molecular alteration in the high-risk group and the low-risk group against previously annotated gene sets (Subramanian et al., 2005). Samples from TCGA-THCA dataset were divided into the high-risk group and the low-risk group according to the optimal cutoff value determined by X-Tile. Thereafter, GSEA was run on javaGSEA v4.0.3 based on the Molecular Signatures Database v7.1. H: Hallmark gene sets, C2: curated gene sets and C5: gene otology gene sets were searched to identify enriched Kyoto Encyclopedia of Genes and Genomes (KEGG) pathways, biological processes, cellular components, molecular functions, and specific well-defined biological states or processes associated with poorer survival of the high-risk group. Gene sets with |NES| >1 and FDR <0.25 were considered statistically significant.

### Validation of the Independent Prognostic Role of the DE-mRNA Signature

To validate the independent prognostic role of the DE-mRNA signature, univariate and multivariate Cox regression analyses were performed on the prognostic DE-mRNA signature and clinical parameters, including age; PFI status; mutational status of *BRAF* V600E, *RAS, RET, NTRK1*, and *TERT*; sex; histological subtype; T stage; N stage; M stage; AJCC stage; residual tumor status; extrathyroidal extension; multifocality; and anatomic site in TCGA dataset. Parameters with *P* < 0.25 in univariate analysis were further included in the multivariate Cox regression analysis. *P* < 0.05 was considered statistically significant.

### Building and Validation of a Prognostic Nomogram

Following a test for collinearity, independent prognostic parameters and relevant clinical parameters were included to construct a prognostic nomogram to predict 1-, 2-, 3-, 4-, and 5-year PFI of PTC patients in the entire TCGA dataset with the stepwise Cox regression model. Discrimination of the nomogram was assessed with the C-index calculated by a bootstrap method with 1,000 resamples. The predicted progression-free survival of nomogram against observed survival rates was plotted using the calibration curve. The performance of the prognostic nomogram was further assessed with ROC curves and Kaplan–Meier analysis. The ATA risk stratification system, MACIS score, and AJCC staging system were used as references for comparison of AUCs. Information of ATA risk stratification and MACIS score for each case was obtained from the official TCGA publication (Cancer Genome Atlas Research, 2014).

### Statistical Analysis

Statistical analysis was conducted using R software v3.4.3 (https://www.r-project.org/), and GraphPad Prism v8.01 (https://www.graphpad.com/). Categorical variables were analyzed with χ^2^-test or Fisher exact test. Continuous variables of two groups were analyzed with Student *t*-test. Multiple groups of continuous variables were analyzed with a one-way analysis of variance test. DE-mRNAs associated with progression-free survival were identified based on univariate and multivariate Cox regression analysis. Hazard ratio (HR) and 95% confidence interval (CI) were calculated. Correlation between two variables was analyzed with Pearson correlation coefficient. ROC curves were analyzed using the “timeROC” R package, and the AUCs were compared with the method described by Blanche et al. (2013). A two-tailed *P* < 0.05 was considered statistically significant if there was no special statement.

## Results

### Identification of DE-circRNAs, DE-miRNAs, and DE-mRNAs in PTC

**Figure 1:**
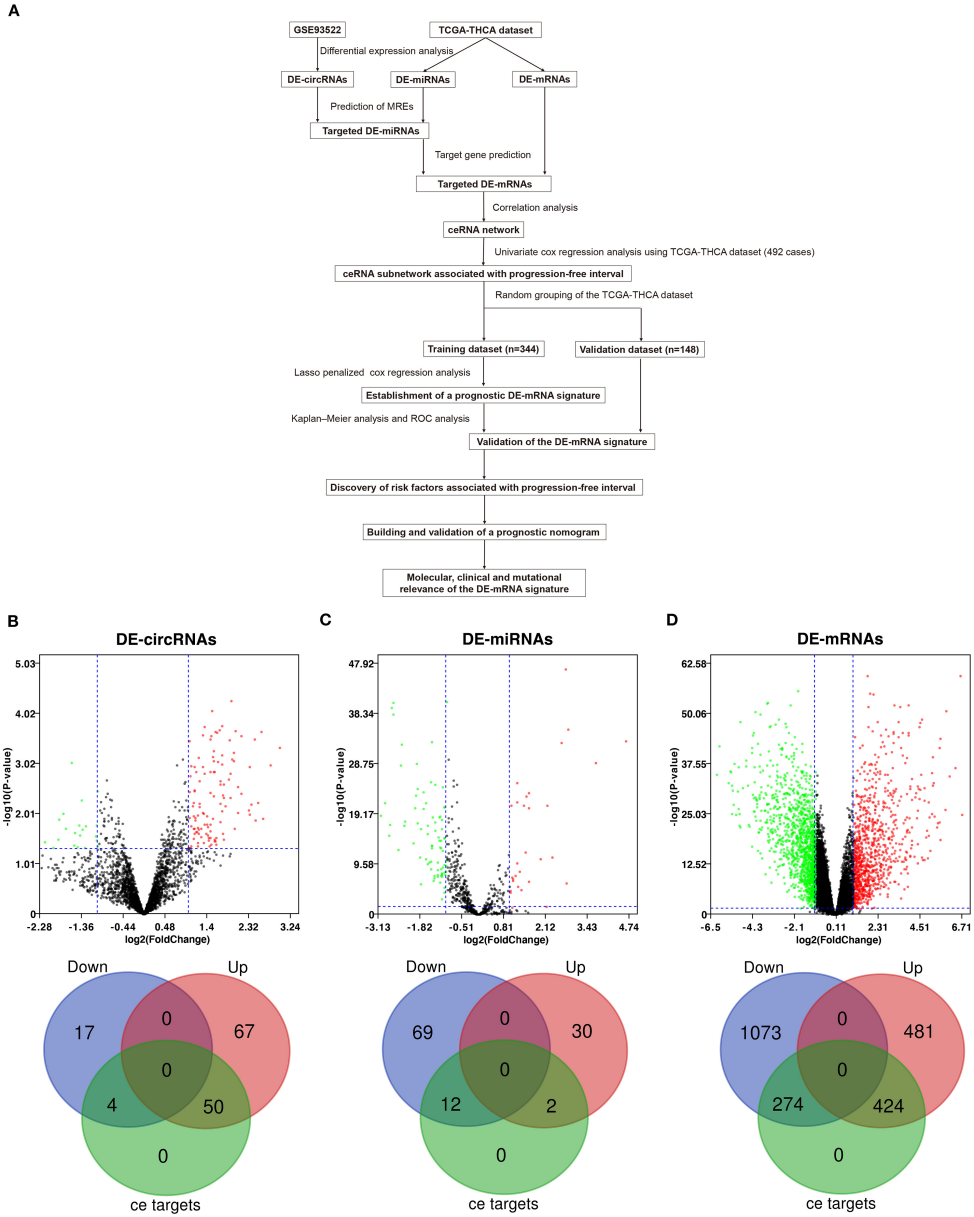
Differential expression analysis and prediction of potential circRNA-associated competing endogenous RNA (ceRNA) regulatory relationships. **(A)** The workflow of constructing a circRNA-associated ceRNA network and the establishment of novel prognostic DE-mRNA signature and nomogram to predict risk of progression in post-surgical papillary thyroid carcinoma (PTC). **(B)** The volcano plot and the Venn diagram show that 138 differentially expressed circRNAs (DE-circRNAs) are identified in PTC (117 upregulated and 21 downregulated). Of these, 54 DE-circRNAs are associated with potential ceRNA regulatory relationships. **(C)** The volcano plot and the Venn diagram show that 113 differentially expressed miRNAs (DE-miRNAs) are identified in PTC (32 upregulated and 81 downregulated). Of these, 14 DE-miRNAs are associated with potential ceRNA regulatory relationships. **(D)** The volcano plot and the Venn diagram show that 2,252 differentially expressed mRNAs (DE-mRNAs) were identified in PTC (905 upregulated and 1,347 downregulated). Of these, 968 DE-mRNAs are associated with potential ceRNA regulatory relationships.

Workflow of the current study is shown in Figure 1A. DE-circRNAs, miRNAs, and mRNAs between PTC and normal thyroid tissues were identified after normalization of expression data. A total of 138 DE-circRNAs were obtained from GSE93522, of which 117 DE-circRNAs were upregulated and 21 DE-circRNAs were downregulated (Supplementary Table 1). DE-miRNAs and DE-mRNAs were obtained from TCGA-THCA dataset. Altogether, 113 DE-miRNAs and 2,252 DE-mRNAs were identified (Supplementary Tables 2, 3). Among these, 32 DE-miRNAs and 905 DE-mRNAs were upregulated, and 81 DE-miRNAs and 1347 DE-mRNAs were downregulated.

### Prediction of Potential Circular RNA-Associated ceRNA Regulatory Relationships and Functional Enrichment Analysis

circRNA-miRNA relationships were predicted using Circular RNA Interactome. miRNA–mRNA relationships were predicted using mirWalk 3.0. Based on the targeted relationship combined with differential expression data, 93 potential circRNA-miRNA pairs and 1,105 miRNA–mRNA pairs were identified, which involved 54 DE-circRNAs, 14 DE-miRNAs, and 698 DE-mRNAs (Figures 1B–D and Supplementary Tables 4, 5).

**Figure 2:**
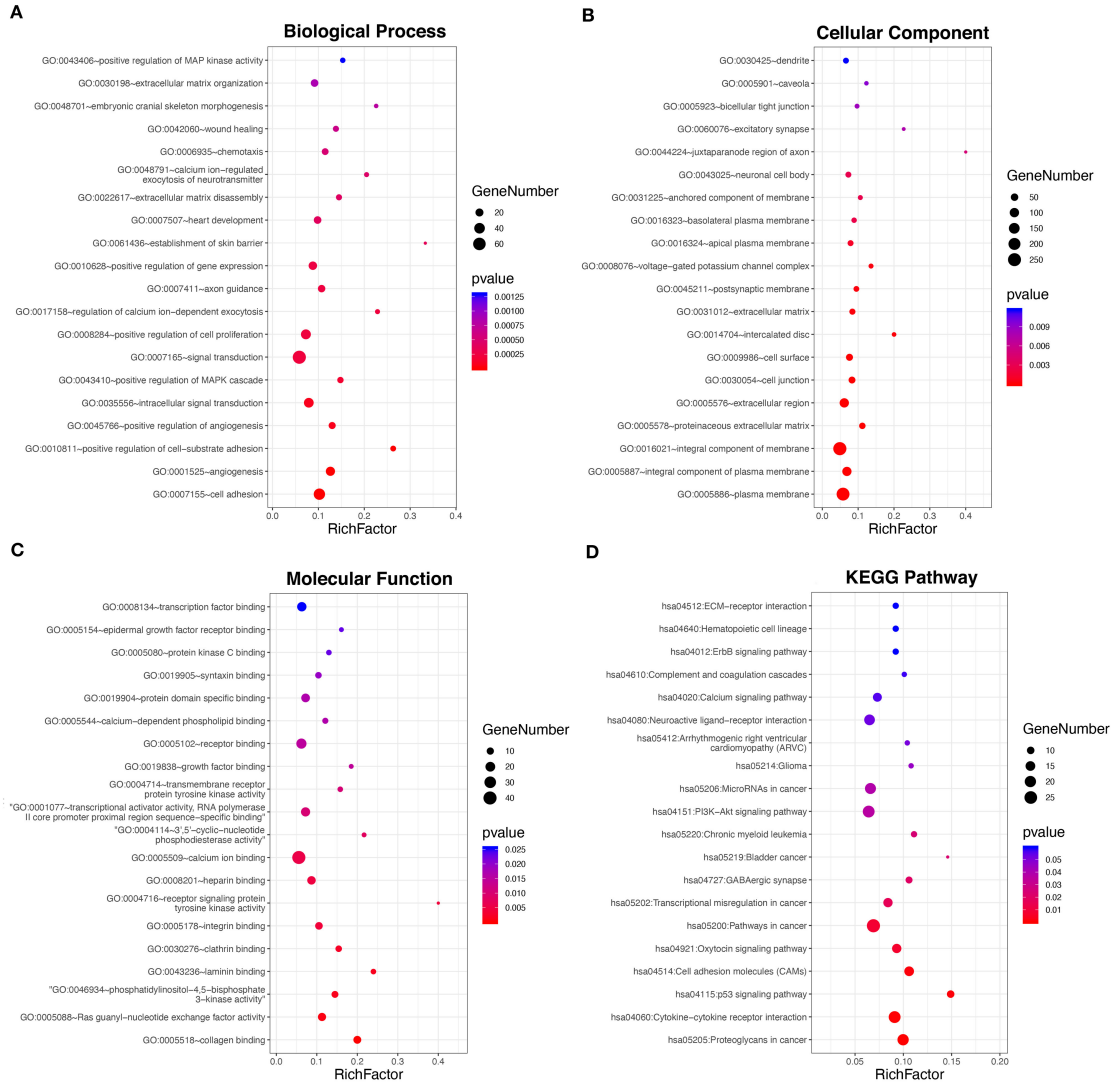
Functional enrichment analysis of the 698 DE-mRNAs with potential circRNA-associated ceRNA regulatory relationships. **(A)** Top 20 most enriched biological processes of the DE-mRNAs. **(B)** Top 20 most enriched cellular components of the DE-mRNAs. **(C)** Top 20 most enriched molecular functions of the DE-mRNAs. **(D)** Top 20 most enriched pathways of the DE-mRNAs.

To investigate the underlying biological function of ceRNA regulatory relationships in PTC, functional enrichment analysis was performed based on the 698 DE-mRNAs involved (Figures 2A–D and Supplementary Table 6). Biological processes associated with malignant phenotype were significantly enriched, which included regulation of cell adhesion, extracellular matrix disassembly and organization, positive regulation of cell proliferation, and angiogenesis. Processes associated with intracellular signal transduction were also significantly enriched, particularly the positive regulation of the mitogen-activated protein kinase cascade. In terms of KEGG pathways, DE-mRNAs were significantly associated with transcriptional misregulation and miRNAs in cancer. Multiple tumor-associated signaling pathways were also enriched, including p53 and PI3K-Akt signaling pathways.

### Construction of the circRNA–miRNA–mRNA Network

**Figure 3:**
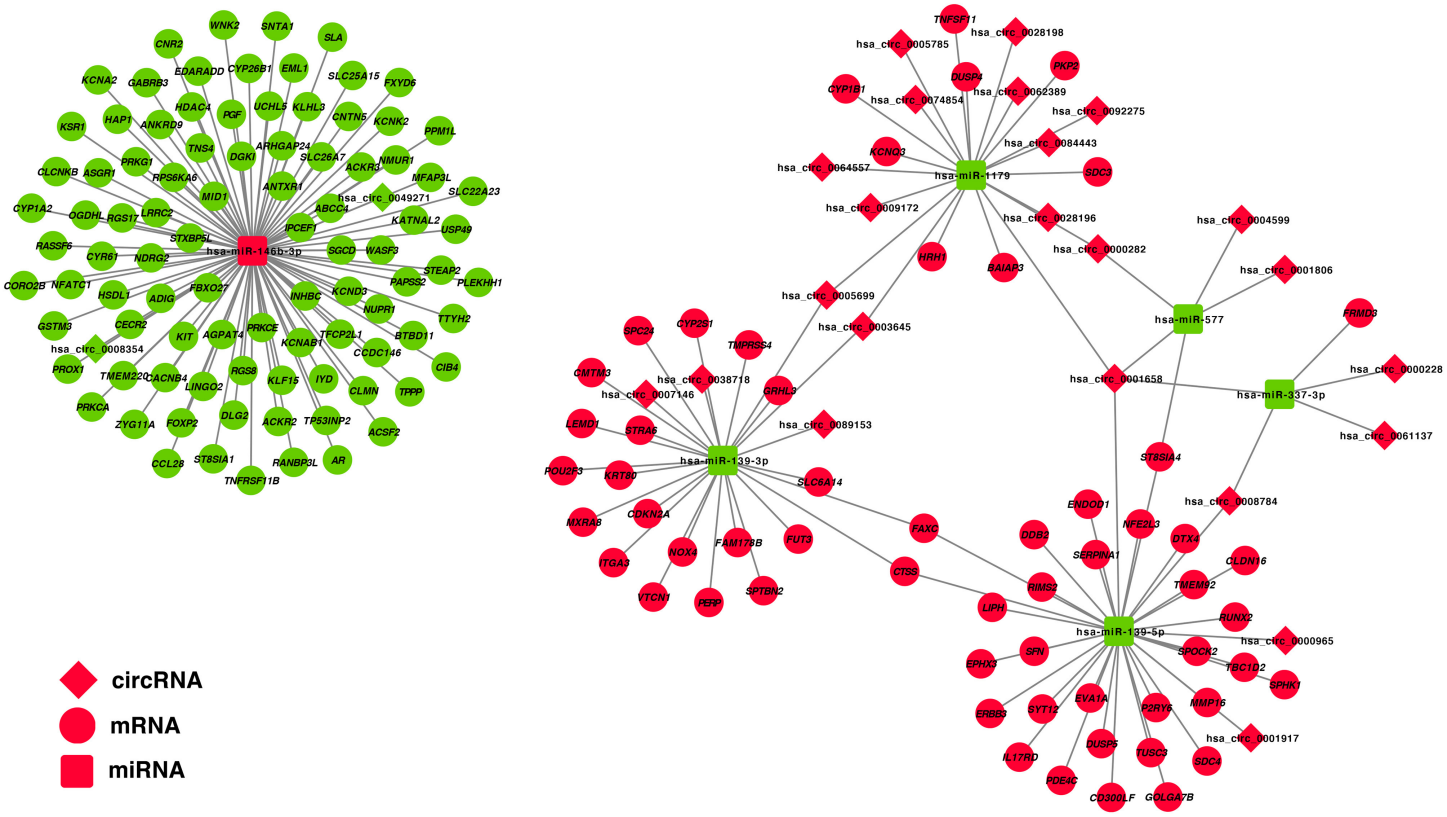
Construction of a circRNA–miRNA–mRNA ceRNA network in the PTC. DE-circRNAs are presented in the diamond nodes, DE-miRNAs are presented in the rounded square nodes, and the DE-mRNAs are presented in the elliptical nodes. Nodes shown in red indicate upregulation in PTC, whereas nodes shown in green indicate downregulation. The circRNA–miRNA–mRNA ceRNA network in PTC includes 25 DE-circRNAs (hsa_circ_0003645, hsa_circ_0089153, hsa_circ_0005699, hsa_circ_0062389, hsa_circ_0028198, hsa_circ_0092275, hsa_circ_0074854, hsa_circ_0028196, hsa_circ_0001658, hsa_circ_0001806, hsa_circ_0007146, hsa_circ_0005785, hsa_circ_0061137, hsa_circ_0009172, hsa_circ_0004599, hsa_circ_0064557, hsa_circ_0038718, hsa_circ_0008784, hsa_circ_0000228, hsa_circ_0000965, hsa_circ_0084443, hsa_circ_0000282, hsa_circ_0001917, hsa_circ_0008354, and hsa_circ_0049271), 6 DE-miRNAs (hsa-miR-146b-3p, hsa-miR-337-3p, hsa-miR-577, hsa-miR-1179, hsa-miR-139-3p, and hsa-miR-139-5p), and 150 DE-mRNAs.

The correlation between DE-miRNA and DE-mRNA expression with potential ceRNA regulatory relationships in PTC was further analyzed using Pearson correlation coefficient. Only miRNA–mRNA relationships with negatively correlated expression were retained. Correlation analysis of miRNAs and potential target mRNAs in TCGA-THCA dataset are shown in Supplementary Table 7. A ceRNA network was constructed, which included 25 DE-circRNAs, 6 DE-miRNAs, and 150 DE-mRNAs based on 32 circRNA-miRNA pairs and 153 miRNA–mRNA pairs (Figure 3).

**Figure 4:**
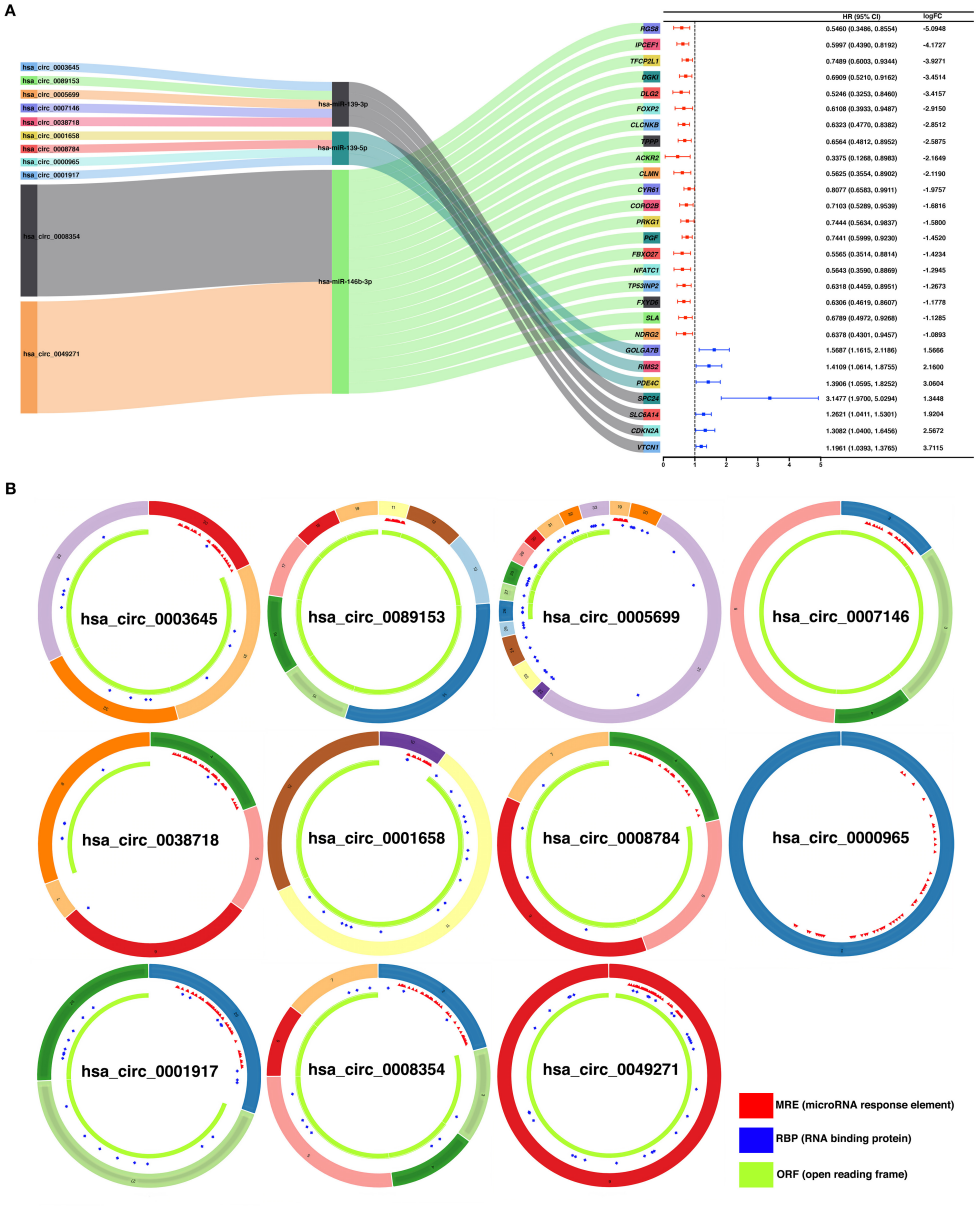
Construction of a ceRNA regulatory subnetwork associated with progression-free interval (PFI) in PTC. **(A)** The Sankey diagram presents the regulatory relationship within the ceRNA regulatory subnetwork associated with PFI in PTC (left). Forest plot of hazard ratio (HR) presenting the prognostic value of the PFI-related DE-mRNAs within the ceRNA subnetwork and the corresponding logFC values for differential expression (PTC vs. normal) (right). **(B)** Basic structures of the 11 DE-circRNAs within the ceRNA subnetwork. The structural patterns of hsa_circ_0003645, hsa_circ_0089153, hsa_circ_0005699, hsa_circ_0007146, hsa_circ_0038718, hsa_circ_0001658, hsa_circ_0008784, hsa_circ_0000965, hsa_circ_0001917, hsa_circ_0008354, and hsa_circ_0049271 provided by Cancer-Specific CircRNA Database (CSCD) are shown. The microRNA response element (MRE) is presented in red. The RNA-binding protein (RBP) is presented in blue and the open reading frame (ORF) is presented in green.

To construct a ceRNA regulatory subnetwork associated with PFI in PTC, univariate Cox regression analysis was performed based on the 150 DE-miRNAs involved. A total of 27 DE-mRNAs were identified to be associated with PFI of PTC. Following retention of the corresponding regulatory circRNA-miRNA and miRNA–mRNA pairs, a ceRNA regulatory subnetwork associated with PFI in PTC was constructed. This included 11 DE-circRNAs, 3 DE-miRNAs and 27 DE-mRNAs based on 11 circRNA-miRNA pairs, and 27 miRNA–mRNA pairs (Figure 4A). hsa_circ_0003645, hsa_circ_0089153, hsa_circ_0005699, hsa_circ_0007146, hsa_circ_0038718, hsa_circ_0001658, hsa_circ_0008784, hsa_circ_0000965, hsa_circ_0001917, hsa_circ_0008354, and hsa_circ_0049271 were DE-circRNAs within the PFI-related subnetwork. The basic characteristics of the 11 DE-circRNAs are shown in Table 1. The corresponding structures of DE-circRNAs were analyzed using the CSCD and shown in Figure 4B.

**Table 1:** Basic characteristics of the 11 differently expressed circRNAs.

**CircRNA ID**	**logFC**	**Regulation**	**Position**	**Strand**	**Spliced length**	**Parental gene symbol**
hsa_circ_0003645	2.46	Up	chr16:19656207-19663412	+	356	*C16orf62*
hsa_circ_0089153	2.37	Up	chr9:134011326-134022971	+	1,102	*NUP214*
hsa_circ_0005699	2.32	Up	chr16:19627435-19663412	+	1,198	*C16orf62*
hsa_circ_0001658	1.57	Up	chr6:157357968-157406039	+	4,8071	*ARID1B*
hsa_circ_0007146	1.48	Up	chr16:5077135-5078186	–	420	*NAGPA*
hsa_circ_0038718	1.22	Up	chr16:27351506-27353580	+	227	*IL4R*
hsa_circ_0008784	1.22	Up	chr13:24164288-24200931	+	479	*TNFRSF19*
hsa_circ_0000965	1.17	Up	chr19:58472758-58476205	–	3,447	*C19orf18*
hsa_circ_0001917	1.02	Up	chrX:41519691-41530783	–	402	*CASK*
hsa_circ_0008354	−1.03	Down	chr19:34981280-34986676	+	485	*WTIP*
hsa_circ_0049271	−1.34	Down	chr19:10610070-10610756	–	686	*KEAP1*

### Identification of Hub PFI-Related DE-mRNAs Regulated by the circRNA-Associated ceRNA Mechanism and Establishment of a Prognostic DE-mRNA Signature

**Figure 5:**
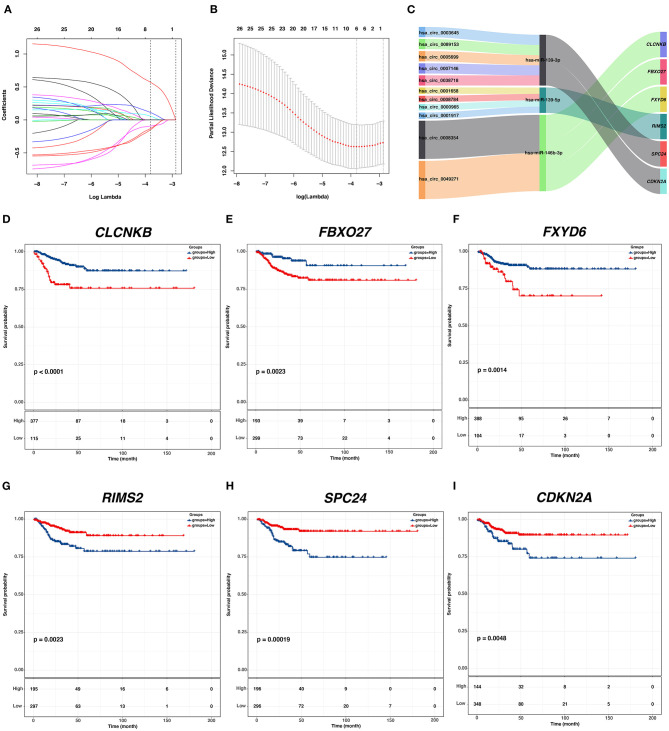
Identification of hub PFI-related DE-mRNAs regulated by ceRNA mechanism and establishment of DE-mRNA signature. **(A)** LASSO coefficient profiles of the 27 prognostic DE-mRNAs within the subnetwork. **(B)** LASSO deviance profiles of the 27 prognostic DE-mRNAs within the subnetwork. **(C)** The Sankey diagram presents the regulatory relationship between the hub PFI-related DE-mRNAs and the corresponding DE-miRNAs and DE-circRNAs. **(D–I)** Kaplan–Meier curves for the hub DE-mRNAs related to PFI based on the optimal cutoff values in PTC. The horizontal axis shows the follow-up time in months, and the vertical axis shows the probability of progression-free survival.

**Figure 6:**
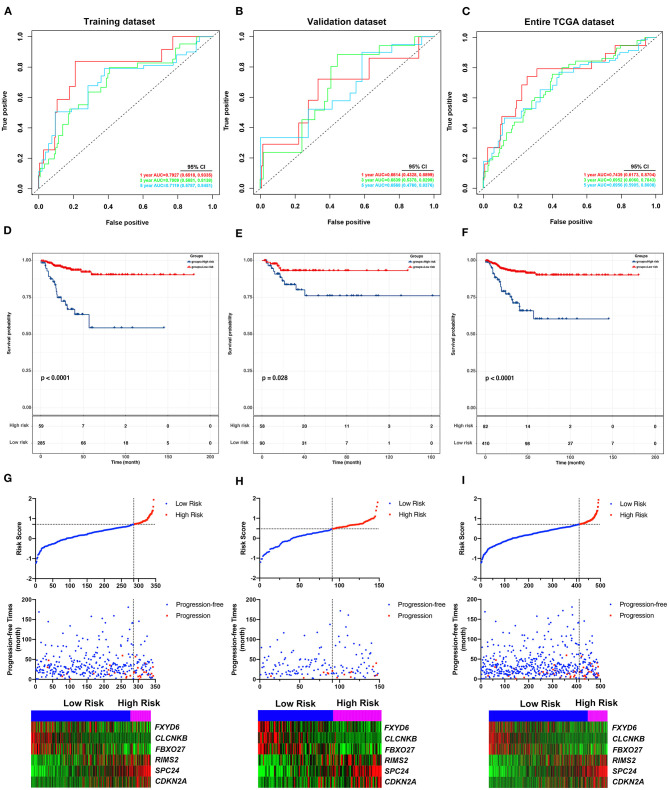
Validation of the predicting performance of the DE-mRNA signature. **(A–C)** The time-dependent receiver operating characteristic (ROC) curves for 1-, 3-, and 5-year PFI predicted by the DE-mRNA signature in the training dataset, the validation dataset, and the entire TCGA-THCA dataset, respectively. **(D–F)** The Kaplan–Meier curves for the DE-mRNA signature based on the optimal cutoff value in the training dataset, the validation dataset, and the entire TCGA-THCA dataset, respectively. The horizontal axis shows the follow-up time in months, and the vertical axis shows the probability of progression-free survival. **(G–I)** The trends in expression profiles of the six hub DE-mRNAs (bottom) and the deterioration of prognosis (middle), along with the incremental risk of progression based on the DE-mRNA signature (upper) in the training dataset, the validation dataset, and the entire TCGA-THCA dataset, respectively.

To identify hub PFI-related DE-mRNAs within the ceRNA subnetwork, 492 TCGA cases with follow-up >30 days were randomly divided into the training dataset and the validation dataset in a 7:3 ratio (Table 2). The training dataset was employed to identify hub DE-mRNAs related to PFI using LASSO–Cox regression. Subsequently, six DE-mRNAs, including *CLCNKB, FXBO27, FXYD6, RIMS2, SPC24*, and *CDKN2A*, were identified as hub PFI-related DE-mRNAs (Figures 5A,B). ceRNA regulatory relationships of the six hub DE-mRNAs are shown in Figure 5C. Downregulated *CLCNKB, FBXO27*, and *FXYD6* with HR <1 were negatively regulated by hsa-miR-146b-3p. Upregulated *RIMS2* with HR >1 was negatively regulated by hsa-miR-139-5p. Moreover, upregulated *SPC24* and *CDKN2A* with HR >1 were negatively regulated by hsa-miR-139-3p. Kaplan–Meier curves for the six hub PFI-related DE-mRNAs based on the optimal cutoff values in PTC are shown in Figures 5D–I. The differential expression of the hub DE-mRNAs between PTC and normal thyroid tissues was validated using GEPIA (Supplementary Figure 1A), and differential expression of corresponding DE-circRNAs and DE-miRNAs is shown in Supplementary Figures 1B–E. A DE-mRNA signature based on the LASSO–Cox regression model coefficients (β) multiplied with corresponding normalized mRNA expression level was established. The risk score was equal to –[(0.09231) ^*^ expression value of *CLCNKB*] – [(0.14778) ^*^ expression value of *FBXO27*] – [(0.12888) ^*^ expression value of *FXYD6*] + [(0.11113) ^*^ expression value of *RIMS2*] + [(0.56913) ^*^ expression value of *SPC24*] + [(0.05512) ^*^ expression value of *CDKN2A*]. The risk score of each case was calculated with this formula. The C-index of the DE-mRNA signature in predicting PFI of PTC was 0.741 (95% CI, 0.564–0.918). ROC curves revealed that the AUCs of the DE-mRNA signature in predicting 1-, 2-, 3-, 4-, and 5-year PFI were 0.793 (95% CI, 0.652–0.934), 0.769 (95% CI, 0.676–0.863), 0.701 (95% CI, 0.588–0.814), 0.687 (95% CI, 0.543–0.831), and 0.712 (95% CI, 0.579–0.845), respectively (Figure 6A). Thereafter, patients were divided into a high-risk and a low-risk group according to the optimal cutoff value determined by X-tile. Kaplan–Meier analysis revealed that high-risk patients with significantly poorer prognosis could be discriminated by the DE-mRNA signature (Figure 6D). As the incremental increase of risk score and the deterioration of prognosis occurred, the expression of *FXYD6, CLCNKB*, and *FBXO27* gradually decreased, and the expression of *RIMS2, SPC24*, and *CDKN2A* gradually increased (Figure 6G). In general, the prognostic DE-mRNA signature based on hub PFI-related DE-mRNAs within the ceRNA subnetwork was accurate in predicting PFI of PTC in the training dataset.

**Table 2:** Clinical features of patients in TCGA-THCA dataset.

**Clinical features**	**Training dataset**	**Validation dataset**	**Entire TCGA dataset**	***P*-value**
*n*	344	148	492	
Follow-up time	1,222.17 ± 996.14	1,237.36 ± 997.68	1,226.74 ± 995.61	0.988
DE-mRNA signature	0.27 ± 0.49	0.24 ± 0.54	0.26 ± 0.50	0.816
Age (y)	46.63 ± 16.08	48.72 ± 15.26	47.26 ± 15.85	0.407
**PFI**				0.996
Progression-free	310 (90.12%)	133 (89.86%)	443 (90.04%)	
Progression	34 (9.88%)	15 (10.14%)	49 (9.96%)	
***BRAF V600E***				0.996
Wild type	132 (38.37%)	55 (37.16%)	187 (38.01%)	
Mutant	201 (58.43%)	89 (60.14%)	290 (58.94%)	
NA	11 (3.20%)	4 (2.70%)	15 (3.05%)	
***RAS*** **mutation**				0.995
Wild type	290 (84.30%)	127 (85.81%)	417 (84.76%)	
Mutant	43 (12.50%)	17 (11.49%)	60 (12.20%)	
NA	11 (3.20%)	4 (2.70%)	15 (3.05%)	
***RET*** **mutation**				0.997
Wild type	310 (90.12%)	135 (91.22%)	445 (90.45%)	
Mutant	23 (6.69%)	9 (6.08%)	32 (6.50%)	
NA	11 (3.20%)	4 (2.70%)	15 (3.05%)	
***NTRK1*** **mutation**				0.606
Wild type	327 (95.06%)	144 (97.30%)	471 (95.73%)	
Mutant	6 (1.74%)	0 (0.00%)	6 (1.22%)	
NA	11 (3.20%)	4 (2.70%)	15 (3.05%)	
***TERT*** **mutation**				0.989
Wild type	330 (95.93%)	142 (95.95%)	472 (95.93%)	
Mutant	3 (0.87%)	2 (1.35%)	5 (1.02%)	
NA	11 (3.20%)	4 (2.70%)	15 (3.05%)	
**Sex**				0.998
Male	92 (26.74%)	40 (27.03%)	132 (26.83%)	
Female	252 (73.26%)	108 (72.97%)	360 (73.17%)	
**Histological type**				0.989
Thyroid papillary carcinoma—classical/usual	247 (71.80%)	103 (69.59%)	350 (71.14%)	
Thyroid papillary carcinoma—follicular (≥99% follicular patterned)	67 (19.48%)	34 (22.97%)	101 (20.53%)	
Thyroid papillary carcinoma—tall cell (≥50% tall cell features)	25 (7.27%)	9 (6.08%)	34 (6.91%)	
Others	5 (1.45%)	2 (1.35%)	7 (1.42%)	
**T**				0.989
T1	99 (28.78%)	43 (29.05%)	142 (28.86%)	
T2	117 (34.01%)	44 (29.73%)	161 (32.72%)	
T3	112 (32.56%)	55 (37.16%)	167 (33.94%)	
T4	15 (4.36%)	6 (4.05%)	21 (4.27%)	
NA	1 (0.29%)	0 (0.00%)	1 (0.20%)	
**N**				0.526
N0	161 (46.80%)	63 (42.57%)	224 (45.53%)	
N1	45 (13.08%)	13 (8.78%)	58 (11.79%)	
N1a	55 (15.99%)	33 (22.30%)	88 (17.89%)	
N1b	45 (13.08%)	27 (18.24%)	72 (14.63%)	
NA	38 (11.05%)	12 (8.11%)	50 (10.16%)	
**M**				0.573
M0 and Mx	199 (57.85%)	78 (52.70%)	277 (56.30%)	
M1	145 (42.15%)	70 (47.30%)	215 (43.70%)	
**AJCC stage**				0.519
Stage I	207 (60.17%)	72 (48.65%)	279 (56.71%)	
Stage II	35 (10.17%)	15 (10.14%)	50 (10.16%)	
Stage III	66 (19.19%)	42 (28.38%)	108 (21.95%)	
Stage IV	35 (10.17%)	18 (12.16%)	53 (10.77%)	
NA	1 (0.29%)	1 (0.68%)	2 (0.41%)	
**Residual tumor**				0.922
R0	258 (75.00%)	117 (79.05%)	375 (76.22%)	
Rx	48 (13.95%)	14 (9.46%)	62 (12.60%)	
R1	35 (10.17%)	16 (10.81%)	51 (10.37%)	
R2	3 (0.87%)	1 (0.68%)	4 (0.81%)	
**Extrathyroid extension**				0.997
None	230 (66.86%)	95 (64.19%)	325 (66.06%)	
Minimal (T3)	90 (26.16%)	42 (28.38%)	132 (26.83%)	
Moderate or advanced (T4)	11 (3.20%)	6 (4.05%)	17 (3.46%)	
NA	13 (3.78%)	5 (3.38%)	18 (3.66%)	
**Multifocality**				0.994
Unifocal	178 (51.74%)	80 (54.05%)	258 (52.44%)	
Multifocal	159 (46.22%)	65 (43.92%)	224 (45.53%)	
NA	7 (2.03%)	3 (2.03%)	10 (2.03%)	
**Anatomic site**				0.845
Unilateral	267 (77.62%)	113 (76.35%)	380 (77.24%)	
Isthmus	14 (4.07%)	8 (5.41%)	22 (4.47%)	
Bilateral	61 (17.73%)	24 (16.22%)	85 (17.28%)	
NA	2 (0.58%)	3 (2.03%)	5 (1.02%)	

### Validation of the DE-mRNA Signature in Predicting PFI of PTC

The performance of the DE-mRNA signature in predicting PFI of the PTC was validated in the validation dataset and the entire TCGA-THCA dataset. The C-index of the DE-mRNA signature in predicting PFI of PTC was 0.665 (95% CI, 0.391–0.939) and 0.716 (95% CI, 0.565–0.867), respectively. ROC curves revealed that in the validation dataset, AUCs of the DE-mRNA signature in predicting 1-, 2-, 3-, 4-, and 5-year PFI were 0.661 (95% CI, 0.433–0.890), 0.647 (95% CI, 0.512–0.783), 0.684 (95% CI, 0.538–0.830), 0.710 (95% CI, 0.554–0.866), and 0.657 (95% CI, 0.476–0.838), respectively (Figure 6B). Moreover, in the entire TCGA-THCA dataset, AUCs of the DE-mRNA signature in predicting 1-, 2-, 3-, 4-, and 5-year PFI were 0.744 (95% CI, 0.617–0.870), 0.728 (95% CI, 0.649–0.806), 0.695 (95% CI, 0.606–0.784), 0.696 (95% CI, 0.589–0.804), and 0.696 (95% CI, 0.591–0.801), respectively (Figure 6C). Thereafter, patients in each dataset were divided into a high-risk and a low-risk group according to the optimal cutoff values determined by X-tile. Kaplan-Meier analysis revealed that high-risk patients with significantly poorer prognosis could be discriminated by the DE-mRNA signature in both datasets (Figures 6E,F). Moreover, heatmaps revealed that the expression of the six DE-mRNAs gradually changed along with risk score increase and prognosis deterioration (Figures 6H,I). The prognostic DE-mRNA signature based on the hub PFI-related DE-mRNAs within the ceRNA subnetwork was confirmed to be robust in predicting PFI of PTC in the validation dataset and the entire TCGA-THCA dataset.

### Analysis of Molecular, Clinical, and Mutational Relevance of the DE-mRNA Signature

**Figure 7:**
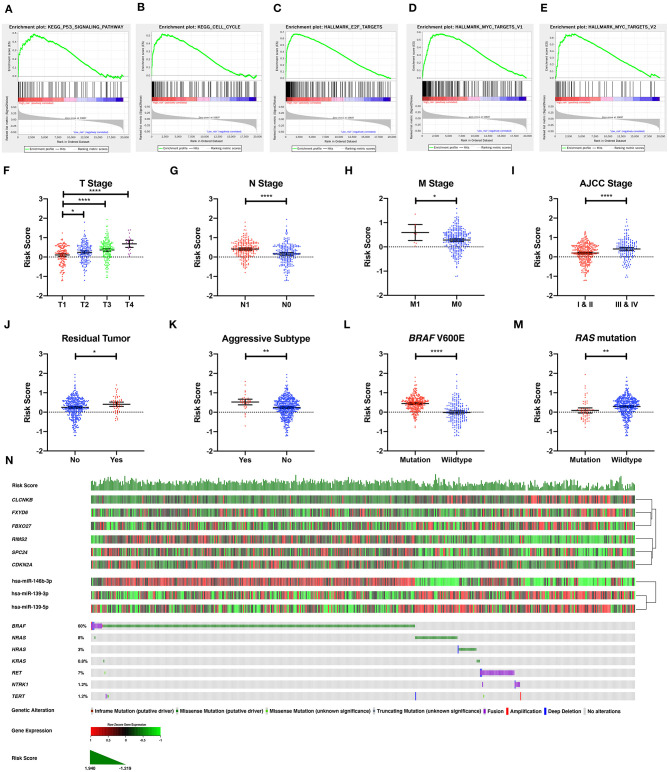
The molecular, clinical, and mutational relevance of the DE-mRNA signature. **(A–E)** Top functional gene sets enriched in the high-risk group predicted by the DE-mRNA signature based on the optimal cutoff values in PTC. **(F)** The distribution of the risk score calculated by the DE-mRNA signature in different groups of T stages in the TCGA-THCA dataset. **(G)** The distribution of the risk score calculated by the DE-mRNA signature in different groups of N stages in the TCGA-THCA dataset. **(H)** The distribution of the risk score calculated by the DE-mRNA signature in different groups of M stages in the TCGA-THCA dataset. **(I)** The distribution of the risk score calculated by the DE-mRNA signature in different groups of AJCC stages in the TCGA-THCA dataset. **(J)** The distribution of the risk score calculated by the DE-mRNA signature in different groups of residual tumor statuses in the TCGA-THCA dataset. **(K)** The distribution of the risk score calculated by the DE-mRNA signature in groups of aggressive and non-aggressive subtypes in the TCGA-THCA dataset. **(L)** The distribution of the risk score calculated by the DE-mRNA signature in different groups of *BRAF* V600E mutation status in the TCGA-THCA dataset. **(M)** The distribution of the risk score calculated by the DE-mRNA signature in different groups of *RAS* mutation status in the TCGA-THCA dataset. **(N)** The relationship among the DE-mRNA signature, the expression profiles of the six hub DE-mRNAs (*CLCNKB, FXYD6, FBXO27, RIMS2, SPC24*, and *CDKN2A*) and corresponding DE-miRNAs (hsa-miR-146b-3p, hsa-miR-139-3p, and hsa-miR-139-5p) and the mutational profiles (*BRAF, RAS, RET, NTRK1*, and *TERT*) of PTC. Data of genetic alteration were obtained from the cBioPortal for Cancer Genomics (https://www.cbioportal.org). *P < 0.05, **P < 0.01, and ****P < 0.0001.

To explore the underlying molecular mechanism of the DE-mRNA signature, the entire TCGA-THCA dataset was divided into a high-risk and a low-risk group based the optimal cutoff value of the DE-mRNA signature determined by X-tile. GSEAs were utilized to compare molecular alteration in the high-risk group and the low-risk group against previously annotated gene sets in the Molecular Signatures Database v7.1. Regarding KEGG pathways, molecular alteration in the high-risk group was significantly enriched in the p53 signaling pathway, cell cycle, etc. (Figures 7A,B). Analysis of hallmark gene sets further revealed that molecular alteration in the high-risk group was significantly associated with genes encoding cell cycle–related targets of E2F transcription factors (Figure 7C). HALLMARK_MYC_TARGETS_V1 and HALLMARK_MYC_TARGETS_V2 was also significantly enriched indicating that the molecular alteration in the high-risk group was potentially regulated by *MYC* (Figures 7D,E). Full results of the GSEA are shown in Supplementary Table 8.

Analysis of clinical relevance revealed that the risk score increased significantly along with the escalation of T stage (*P* < 0.05; Figure 7F). Patients with lymph node and distal metastasis had a significantly higher risk score (*P* < 0.05; Figures 7G,H). Regarding AJCC stage, advanced stages (III and IV) were associated with a higher risk score than early stages (I and II) (*P* < 0.05; Figure 7I). Patients with microscopic and macroscopic residual tumors also had a significantly higher risk score, further indicating the relevance of the DE-mRNA signature to the aggressiveness of PTC (*P* < 0.05; Figure 7J). Analysis of histological subtypes revealed that aggressive subtypes of PTC, including tall cell, hobnail variant, and columnar cell carcinoma, were associated with a significantly higher risk score (*P* < 0.05; Figure 7K).

Analysis of mutational relevance revealed that the *BRAF* V600E mutation and *RAS* mutation was associated with a significantly higher and lower risk score, respectively (*P* < 0.05; Figures 7L,M). The mutational pattern of PTC further revealed that *CLCNKB, FBXO27*, and *FXYD6* had a relatively lower expression level in samples with the *BRAF* V600E mutation and were negatively associated with hsa-miR-146b-3p expression (Figure 7N). Alternatively, *SPC24* and *CDKN2A* negatively regulated by hsa-miR-139-3p had a relatively higher expression level in samples with the *BRAF* V600E mutation. *RIMS2* also had a relatively higher expression level in samples with the *BRAF* V600E mutation and was negatively associated with hsa-miR-139-5p expression. Regarding the *RAS* mutation, the six DE-mRNAs and the corresponding miRNA regulators had opposite expression patterns to the *BRAF* V600E mutation.

### Validation of the Independent Prognostic Role of the DE-mRNA Signature in PTC

A total of 389 cases from the entire TCGA-THCA dataset with complete clinical information were utilized to identify prognostic factors associated with PFI in PTC. Clinical information for analysis included DE-mRNA signature, age, PFI status, mutational status of *BRAF* V600E, *RAS, RET, NTRK1*, and *TERT*, sex, histological subtype, T stage, N stage, M stage, AJCC stage, residual tumor status, extrathyroidal extension, multifocality, and anatomic site. Reasons for exclusion are listed in Supplementary Table 9. Baseline characteristics of patients who were included for prognostic factor evaluation are shown in Table 3. Patients in the high-risk group had significantly worse prognosis; higher T stage, N stage, M stage, and AJCC stage; and a larger portion with the *BRAF* V600E mutation and extrathyroidal extension. Tumors of the high-risk group were also more likely to be unifocal. Univariate Cox regression analysis revealed that DE-mRNA signature, age, histological type, aggressive subtype, T stage, N stage, AJCC stage, and neoplasm largest dimension were prognostic factors associated with PFI of PTC (*P* < 0.05; Table 4). Adjustment for parameters with *P* < 0.25 based on univariate analysis and multivariate Cox regression analysis further validated that the DE-mRNA signature was an independent prognostic factor associated with PFI in PTC (Table 5). The *RAS* mutation was also identified to be an independent prognostic factor.

**Table 3:** Baseline characteristics of patients included for the evaluation of prognostic factors and establishment of nomogram.

**Clinical features**	**Low risk**	**High risk**	***P*-value**
	**Mean** **±*****SD***		
Follow-up time (day)	1,224.96 ± 968.27	1,297.97 ± 925.63	0.570
DE-mRNA signature	0.18 ± 0.37	0.97 ± 0.27	<0.001
Age	46.54 ± 14.69	49.93 ± 18.9	0.102
	*n* (%)		
**PFI**			<0.001
Progression-free	300 (93.46%)	49 (72.06%)	
Progression	21 (6.54%)	19 (27.94%)	
***BRAF V600E***			0.026
Wild type	121 (37.69%)	16 (23.53%)	
Mutant	200 (62.31%)	52 (76.47%)	
***RAS*** **mutation**			0.364
Wild type	280 (87.23%)	62 (91.18%)	
Mutant	41 (12.77%)	6 (8.82%)	
***RET*** **mutation**			0.293
Wild type	295 (91.90%)	65 (95.59%)	
Mutant	26 (8.10%)	3 (4.41%)	
***NTRK1*** **mutation**			0.256
Wild type	315 (98.13%)	68 (100.00%)	
Mutant	6 (1.87%)	0 (0.00%)	
***TERT*** **mutation**			0.182
Wild type	318 (99.07%)	66 (97.06%)	
Mutant	3 (0.93%)	2 (2.94%)	
**Sex**			0.180
Male	83 (25.86%)	23 (33.82%)	
Female	238 (74.14%)	45 (66.18%)	
**Histological type**			0.303
Thyroid papillary carcinoma—classical/usual	226 (70.40%)	52 (76.47%)	
Thyroid papillary carcinoma—follicular (≥99% follicular patterned)	67 (20.87%)	8 (11.76%)	
Thyroid papillary carcinoma—tall cell (≥50% tall cell features)	22 (6.85%)	7 (10.29%)	
Others	6 (1.87%)	1 (1.47%)	
**T**			<0.001
T1	107 (33.33%)	6 (8.82%)	
T2	104 (32.40%)	20 (29.41%)	
T3	103 (32.09%)	32 (47.06%)	
T4	7 (2.18%)	10 (14.71%)	
**N**			<0.001
N0	175 (54.52%)	18 (26.47%)	
N1	39 (12.15%)	16 (23.53%)	
N1a	63 (19.63%)	16 (23.53%)	
N1b	44 (13.71%)	18 (26.47%)	
**M**			0.040
M0 and Mx	199 (61.99%)	33 (48.53%)	
M1	122 (38.01%)	35 (51.47%)	
**AJCC stage**			<0.001
Stage I	188 (58.57%)	28 (41.18%)	
Stage II	37 (11.53%)	2 (2.94%)	
Stage III	72 (22.43%)	18 (26.47%)	
Stage IV	24 (7.48%)	20 (29.41%)	
**Residual tumor**			0.073
R0	265 (82.55%)	48 (70.59%)	
Rx	23 (7.17%)	10 (14.71%)	
R1	30 (9.35%)	10 (14.71%)	
R2	3 (0.93%)	0 (0.00%)	
**Extrathyroid extension**			<0.001
None	228 (71.03%)	35 (51.47%)	
Minimal (T3)	87 (27.10%)	25 (36.76%)	
Moderate or advanced (T4)	6 (1.87%)	8 (11.76%)	
**Multifocality**			0.006
Unifocal	163 (50.78%)	47 (69.12%)	
Multifocal	158 (49.22%)	21 (30.88%)	
**Anatomic site**			0.585
Unilateral	248 (77.26%)	56 (82.35%)	
Isthmus	17 (5.30%)	2 (2.94%)	
Bilateral	56 (17.45%)	10 (14.71%)	

**Table 4:** Unadjusted univariate Cox analysis.

**Clinical features**	**Statistics**	**PFI**
DE-mRNA signature	0.32 ± 0.46	6.90 (3.45, 13.79), <0.0001
***BRAF V600E***		
Wild type	137 (35.22%)	1.0
Mutant	252 (64.78%)	1.06 (0.55, 2.06), 0.860
***RAS*** **mutation**		
Wild type	342 (87.92%)	1.0
Mutant	47 (12.08%)	1.72 (0.76, 3.89), 0.194
***TERT*** **mutation**		
Wild type	384 (98.71%)	1.0
Mutant	5 (1.29%)	0.00 (0.00, Inf), 0.997
***RET*** **mutation**		
Wild type	360 (92.54%)	1.0
Mutant	29 (7.46%)	0.72 (0.17, 3.00), 0.657
***NTRK1*** **mutation**		
Wild type	383 (98.46%)	1.0
Mutant	6 (1.54%)	0.00 (0.00, Inf), 0.996
**Sex**		
Male	106 (27.25%)	1.0
Female	283 (72.75%)	0.76 (0.39, 1.47), 0.411
Age	47.13 ± 15.54	1.02 (1.00, 1.04), 0.042
**Age**		
≤55 years	275 (70.69%)	1.0
>55 years	114 (29.31%)	2.36 (1.27, 4.41), 0.007
**Histological type**		
Thyroid papillary carcinoma—classical/usual	278 (71.47%)	1.0
Thyroid papillary carcinoma—follicular (≥99% follicular patterned)	75 (19.28%)	0.72 (0.28, 1.87), 0.501
Thyroid papillary carcinoma—tall cell (≥50% tall cell features)	29 (7.46%)	2.52 (1.04, 6.08), 0.041
Others	7 (1.80%)	0.00 (0.00, Inf), 0.997
**Aggressive subtype**		
No	359 (92.29%)	1.0
Yes	30 (7.71%)	2.70 (1.13, 6.46), 0.025
**T**		
T1	113 (29.05%)	1.0
T2	124 (31.88%)	2.63 (0.85, 8.16), 0.094
T3	135 (34.70%)	4.22 (1.44, 12.36), 0.009
T4	17 (4.37%)	5.73 (1.43, 22.98), 0.014
**N**		
N0	193 (49.61%)	1.0
N1	196 (50.39%)	2.11 (1.09, 4.08), 0.027
**M**		
M0 and Mx	232 (59.64%)	1.0
M1	157 (40.36%)	1.65 (0.89, 3.08), 0.112
**AJCC stage**		
Stage I	216 (55.53%)	1.0
Stage II	39 (10.03%)	1.13 (0.32, 3.93), 0.849
Stage III	90 (23.14%)	2.67 (1.27, 5.60), 0.010
Stage IV	44 (11.31%)	3.89 (1.68, 9.02), 0.002
**Residual tumor**		
R0	313 (80.46%)	1.0
Rx	33 (8.48%)	1.76 (0.61, 5.01), 0.293
R1	40 (10.28%)	1.74 (0.72, 4.18), 0.219
R2	3 (0.77%)	3.23 (0.44, 23.91), 0.251
**Extrathyroid extension**		
None	263 (67.61%)	1.0
Minimal (T3)	112 (28.79%)	1.85 (0.97, 3.50), 0.060
Moderate or advanced (T4)	14 (3.60%)	1.77 (0.42, 7.58), 0.439
Neoplasm largest dimension (cm)	2.80 ± 1.58	1.24 (1.04, 1.46), 0.014
**Neoplasm largest dimension**		
≤2 cm	153 (39.33%)	1.0
>2 cm	236 (60.67%)	3.61 (1.51, 8.61), 0.004
**Multifocality**		
Unifocal	210 (53.98%)	1.0
Multifocal	179 (46.02%)	0.82 (0.43, 1.57), 0.553
**Anatomic site**		
Unilateral	304 (78.15%)	1.0
Isthmus	19 (4.88%)	0.45 (0.06, 3.27), 0.429
Bilateral	66 (16.97%)	0.94 (0.39, 2.26), 0.898

**Table 5:** Multivariate Cox regression analysis.

**Clinical features**	**Non-adjusted**	**Adjust I**	**Adjust II**	**Adjust III**
DE-mRNA signature	6.90 (3.45, 13.79), <0.0001	5.60 (2.73, 11.49), <0.0001	5.60 (2.73, 11.49), <0.0001	5.39 (2.45, 11.85), <0.0001
***BRAF V600E***				
Wild type	1.0	1.0	1.0	NA
Mutant	1.06 (0.55, 2.06), 0.860	0.97 (0.50, 1.90), 0.936	0.63 (0.32, 1.25), 0.183	NA
***RAS*** **mutation**				
Wild type	1.0	1.0	1.0	1.0
Mutant	1.72 (0.76, 3.89), 0.194	2.00 (0.87, 4.57), 0.100	2.42 (1.05, 5.57), 0.037	5.99 (1.88, 19.05), 0.002
***TERT*** **mutation**				
Wild type	1.0	1.0	1.0	NA
Mutant	0.00 (0.00, Inf), 0.997	0.00 (0.00, Inf), 0.997	0.00 (0.00, Inf), 0.997	NA
***RET*** **mutation**				
Wild type	1.0	1.0	1.0	NA
Mutant	0.72 (0.17, 3.00), 0.657	0.69 (0.16, 2.91), 0.611	1.05 (0.24, 4.53), 0.946	NA
***NTRK1*** **mutation**				
Wild type	1.0	1.0	1.0	NA
Mutant	0.00 (0.00, Inf), 0.996	0.00 (0.00, Inf), 0.996	0.00 (0.00, Inf), 0.997	NA
**Sex**				
Male	1.0	1.0	1.0	NA
Female	0.76 (0.39, 1.47), 0.411	0.91 (0.46, 1.81), 0.789	1.00 (0.50, 1.99), 0.998	NA
Age	1.02 (1.00, 1.04), 0.042	1.00 (0.97, 1.03), 0.847	0.99 (0.97, 1.02), 0.618	1.00 (0.97, 1.03), 0.957
**Age**				
≤55 years	1.0	1.0	1.0	1.0
>55 years	2.36 (1.27, 4.41), 0.007	1.46 (0.68, 3.14), 0.331	1.14 (0.49, 2.64), 0.758	1.43 (0.53, 3.81), 0.479
**Histological type**				
Thyroid papillary carcinoma—classical/usual	1.0		1.0	1.0
Thyroid papillary carcinoma—follicular (≥99% follicular patterned)	0.72 (0.28, 1.87), 0.501	0.79 (0.30, 2.08), 0.634	0.91 (0.34, 2.41), 0.846	0.53 (0.15, 1.82), 0.310
Thyroid papillary carcinoma—tall cell (≥50% tall cell features)	2.52 (1.04, 6.08), 0.041	1.91 (0.76, 4.81), 0.172	1.48 (0.58, 3.79), 0.418	0.04 (0.00, Inf), 0.999
Others	0.00 (0.00, Inf), 0.997	0.00 (0.00, Inf), 0.997	0.00 (0.00, Inf), 0.997	0.00 (0.00, Inf), 0.996
**Aggressive subtype**				
No	1.0	1.0	1.0	1.0
Yes	2.70 (1.13, 6.46), 0.025	2.00 (0.80, 4.99), 0.139	1.50 (0.59, 3.83), 0.393	49.47 (0.00, Inf), 0.999
**T**				
T1	1.0	1.0	1.0	1.0
T2	2.63 (0.85, 8.16), 0.094	2.65 (0.81, 8.67), 0.107	2.08 (0.64, 6.78), 0.226	2.29 (0.67, 7.74), 0.184
T3	4.22 (1.44, 12.36), 0.009	3.01 (0.95, 9.56), 0.062	2.41 (0.77, 7.59), 0.132	3.44 (0.77, 15.38), 0.107
T4	5.73 (1.43, 22.98), 0.014	2.47 (0.51, 12.06), 0.263	1.33 (0.27, 6.61), 0.725	1.35 (0.15, 12.00), 0.787
**N**				
N0	1.0	1.0	1.0	1.0
N1	2.11 (1.09, 4.08), 0.027	1.62 (0.76, 3.49), 0.213	1.47 (0.69, 3.13), 0.311	2.38 (0.97, 5.80), 0.057
**M**				
M0 and Mx	1.0	1.0	1.0	1.0
M1	1.65 (0.89, 3.08), 0.112	1.64 (0.87, 3.07), 0.123	1.21 (0.63, 2.35), 0.563	1.20 (0.58, 2.50), 0.622
**AJCC stage**				
Stage I	1.0	1.0	1.0	1.0
Stage II	1.13 (0.32, 3.93), 0.849	1.19 (0.31, 4.59), 0.800	1.61 (0.41, 6.31), 0.492	1.15 (0.19, 7.01), 0.876
Stage III	2.67 (1.27, 5.60), 0.010	2.84 (1.06, 7.57), 0.038	2.48 (0.85, 7.20), 0.096	1.39 (0.42, 4.62), 0.595
Stage IV	3.89 (1.68, 9.02), 0.002	4.05 (1.38, 11.91), 0.011	3.34 (1.06, 10.50), 0.039	2.07 (0.53, 8.07), 0.293
**Residual tumor**				
R0	1.0	1.0	1.0	1.0
Rx	1.76 (0.61, 5.01), 0.293	1.35 (0.47, 3.93), 0.579	1.25 (0.43, 3.64), 0.684	1.56 (0.48, 5.06), 0.462
R1	1.74 (0.72, 4.18), 0.219	1.20 (0.49, 2.96), 0.691	1.14 (0.46, 2.84), 0.776	1.17 (0.44, 3.12), 0.748
R2	3.23 (0.44, 23.91), 0.251	5.58 (0.47, 66.80), 0.174	4.15 (0.33, 52.73), 0.272	11.00 (0.78, 154.44), 0.075
**Extrathyroid extension**				
None	1.0	1.0	1.0	1.0
Minimal (T3)	1.85 (0.97, 3.50), 0.060	1.30 (0.64, 2.61), 0.465	1.23 (0.62, 2.47), 0.553	0.71 (0.24, 2.10), 0.533
Moderate or advanced (T4)	1.77 (0.42, 7.58), 0.439	0.68 (0.14, 3.30), 0.632	0.46 (0.09, 2.23), 0.332	0.83 (0.09, 7.48), 0.865
Neoplasm largest dimension	1.24 (1.04, 1.46), 0.014	1.14 (0.96, 1.36), 0.140	1.04 (0.86, 1.25), 0.686	0.95 (0.76, 1.20), 0.691
**Neoplasm largest dimension**				
≤2 cm	1.0	1.0	1.0	1.0
>2 cm	3.61 (1.51, 8.61), 0.004	3.55 (1.48, 8.52), 0.005	2.69 (1.10, 6.55), 0.030	2.50 (0.76, 8.20), 0.131
**Multifocality**				
Unifocal	1.0	1.0	1.0	NA
Multifocal	0.82 (0.43, 1.57), 0.553	0.72 (0.37, 1.39), 0.322	0.92 (0.47, 1.77), 0.792	NA
**Anatomic site**				
Unilateral	1.0	1.0	1.0	NA
Isthmus	0.45 (0.06, 3.27), 0.429	0.48 (0.06, 3.54), 0.472	0.41 (0.06, 3.00), 0.377	NA
Bilateral	0.94 (0.39, 2.26), 0.898	0.73 (0.30, 1.77), 0.486	0.88 (0.36, 2.14), 0.782	NA

*Adjust I model adjusted for age, sex, and AJCC stage*.

*Adjust II model adjusted for age, sex, AJCC stage, and DE-mRNA signature*.

*Adjust III model adjusted for parameters with P < 0.25 based on univariate analysis*.

### Building and Validation of a DE-mRNA Signature–Based Prognostic Nomogram

**Figure 8:**
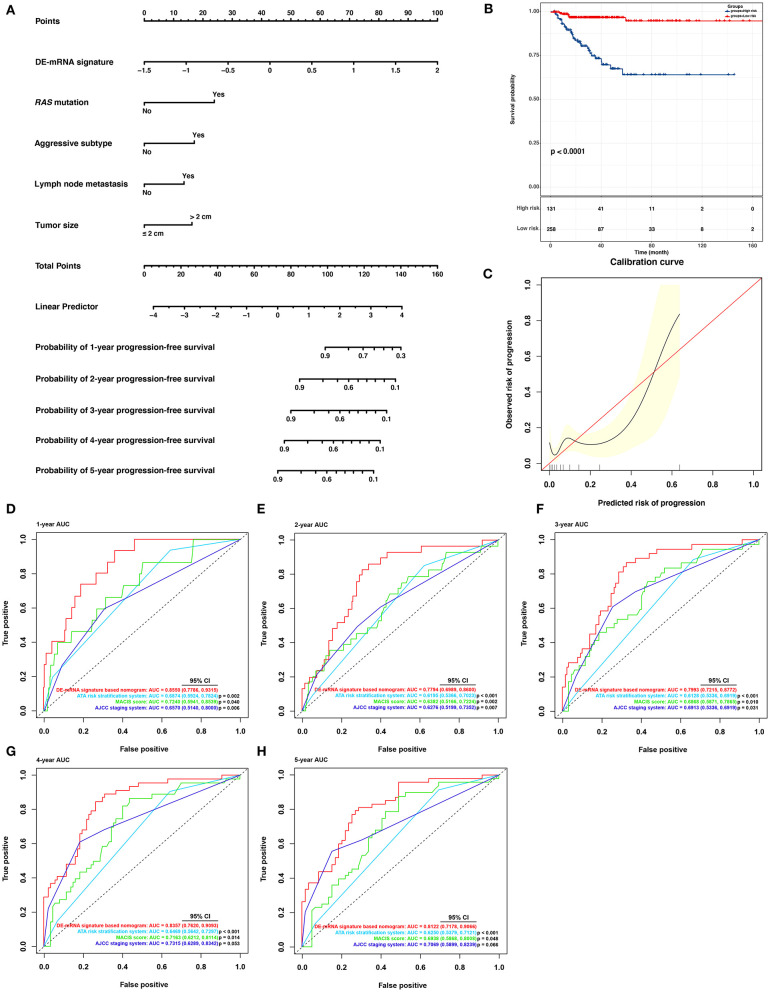
Building and validation of a DE-mRNA signature–based prognostic nomogram predicting PFI in PTC. **(A)** A DE-mRNA signature–based prognostic nomogram predicting 1-, 2-, 3-, 4-, and 5-year PFI in PTC. **(B)** The Kaplan–Meier curves for the nomogram based on the optimal cutoff value in the entire TCGA-THCA dataset. The horizontal axis shows the PFI time in months, and the vertical axis shows the probability of progression-free survival. **(C)** The calibration plot for internal validation of the nomogram. The *X* axis represents the predicted risk of progression, whereas the Y axis represents the observed risk of progression. **(D)** The time-dependent ROC curves for 1-year PFI predicted by the nomogram in comparison with the ATA risk stratification system, the MACIS score, and the AJCC staging system. **(E)** The time-dependent ROC curves for 2-year PFI predicted by the nomogram in comparison with the ATA risk stratification system, the MACIS score, and the AJCC staging system. **(F)** The time-dependent ROC curves for 3-year PFI predicted by the nomogram in comparison with the ATA risk stratification system, the MACIS score, and the AJCC staging system. **(G)** The time-dependent ROC curves for 4-year PFI predicted by the nomogram in comparison with the ATA risk stratification system, the MACIS score, and the AJCC staging system. **(H)** The time-dependent ROC curves for 5-year PFI predicted by the nomogram in comparison with the ATA risk stratification system, the MACIS score, and the AJCC staging system. AUCs were compared with the method described by Subramanian et al. (2005).

Based on cases from the entire TCGA-THCA dataset with complete clinical information, a prognostic nomogram was established with the DE-mRNA signature and prognostic clinical factors using stepwise Cox regression (Figure 8A). Factors involved in the nomogram included the DE-mRNA signature, *RAS* mutation status, aggressive subtype, N stage, and tumor size. The C-index of the nomogram in predicting PFI of PTC was 0.790 (95% CI, 0.652–0.927). The calibration curve revealed that the predicted risk of progression by the nomogram was close to the observed risk of progression (Figure 8C). Patients were subsequently divided into the high-risk and the low-risk group according to the optimal cutoff value determined by X-tile. Kaplan–Meier analysis revealed that high-risk patients had significantly poorer prognosis than low-risk patients (Figure 8B). The ATA risk stratification system is recommended to estimate the risk of post-surgical recurrent disease in PTC. MACIS score and AJCC staging system are employed to predict post-surgical mortality of PTC. The accuracy of nomogram in predicting PFI of PTC was analyzed with ROC curves using the ATA risk stratification system, MACIS score, and AJCC staging system as reference (Figures 8D–H). The AUCs of the nomogram in predicting 1-, 2-, 3-, 4-, and 5-year PFI were 0.855 (95% CI, 0.779–0.932), 0.779 (95% CI, 0.699–0.860), 0.799 (95% CI, 0.722–0.877), 0.836 (95% CI, 0.762–0.909), and 0.812 (95% CI, 0.718–0.907), respectively. The AUCs of the ATA risk stratification system in predicting 1-, 2-, 3-, 4-, and 5-year PFI were 0.687 (95% CI, 0.592–0.782), 0.620 (95% CI, 0.537–0.702), 0.613 (95% CI, 0.534–0.692), 0.647 (95% CI, 0.564–0.730), and 0.625 (95% CI, 0.538–0.712), respectively. The AUCs of the MACIS score in predicting 1-, 2-, 3-, 4-, and 5-year PFI were 0.724 (95% CI, 0.594–0.854), 0.638 (95% CI, 0.517–0.722), 0.687 (95% CI, 0.587–0.787), 0.716 (95% CI, 0.621–0.811), and 0.694 (95% CI, 0.587–0.801), respectively. The AUCs of the AJCC staging system in predicting 1-, 2-, 3-, 4-, and 5-year PFI were 0.657 (95% CI, 0.514–0.800), 0.628 (95% CI, 0.520–0.735), 0.691 (95% CI, 0.534–0.692), 0.732 (95% CI, 0.629–0.834), and 0.707 (95% CI, 0.590–0.824), respectively. The nomogram had significantly higher AUCs in predicting 1-, 2-, 3-, 4-, and 5-year PFI in comparison with the ATA risk stratification system and MACIS score and had significantly higher AUCs in predicting 1-, 2-, and 3-year PFI in comparison with the AJCC staging system (*P* < 0.05).

## Discussion

Accumulating evidence has shown that circRNAs and their mediated ceRNA regulatory networks play a vital role in pathogenesis and progression of various human cancers, including PTC. hsa-circ-0058124 was previously identified to be upregulated in PTC and associated with poor prognosis. By sponging hsa-miR-218-5p, hsa-circ-0058124 promoted proliferation, invasion, and metastasis of PTC via the *NOTCH3*/*GATAD2A* signaling axis (Yao et al., 2019). Through regulation of *ABCA9* and *MTA1* via sponge hsa-miR-1179 and hsa-miR-1205, upregulated hsa_circ_0039411 promoted proliferation, migration, and invasion of PTC cells and inhibited cell apoptosis (Yang et al., 2020). CircRNAs are resistant to exonase and RNase R, enabling them to be more stable than other types of ncRNAs, as well as play an important regulatory role in cancer cell. The current understanding of the circRNA-related ceRNA network in PTC is limited, requiring further study.

In this study, DE-circRNAs, DE-miRNAs, and DE-mRNAs with potential ceRNA regulatory relationships in PTC were identified through comprehensive analysis of targeting relationship prediction and differential expression data. Based on the further correlation analysis of expression between DE-miRNAs and DE-mRNAs with potential ceRNA regulatory relationships, a circRNA–miRNA–mRNA regulatory network was constructed in PTC. DE-mRNAs within the ceRNA network associated with PFI of PTC were further identified to construct a ceRNA regulatory subnetwork associated with PFI. Six hub DE-mRNAs, namely, *CLCNKB, FXBO27, FXYD6, RIMS2, SPC24*, and *CDKN2A*, were identified to be most significantly related to the PFI of PTC, which were regulated by three DE-miRNAs, including hsa-miR-146b-3p, hsa-miR-139-5p, and hsa-miR-139-3p. They were subsequently regulated by 11 DE-circRNAs via the ceRNA mechanism, including hsa_circ_0003645, hsa_circ_0089153, hsa_circ_0005699, hsa_circ_0007146, hsa_circ_0038718, hsa_circ_0001658, hsa_circ_0008784, hsa_circ_0000965, hsa_circ_0001917, hsa_circ_0008354, and hsa_circ_0049271. These newly identified key circRNA–miRNA–mRNA regulatory relationships may help elucidate the molecular mechanism of progression in post-surgical PTC. Such insights may provide novel therapeutic targets for treatment of recurrence in PTC. Hub DE-mRNAs identified in the ceRNA network were potential predictors of PFI in PTC.

Although most patients with PTC have a relatively good prognosis, a portion of patients will eventually develop post-surgical recurrence. These high-risk PTC patients should be distinguished early enough to adopt more aggressive treatment options, additional adjuvant radioactive iodine therapy, thyroid hormone suppression therapy with a higher dosage, and timely intervention to prevent post-surgical progression if necessary. In contrast, for the remaining low-risk PTC patients, aggressive treatment options should be avoided to improve quality of life. Therefore, it is critical to accurately predict the post-surgical risk of progression in patients with PTC. Currently, the ATA guidelines recommend the use of AJCC staging system and MACIS score to predict the risk of post-operative mortality. ATA risk stratification system was recommended to assess post-surgical risk of recurrence. The existing risk assessment tools are not sufficiently accurate, and a novel prediction system should be established to more accurately predict the prognosis of PTC patients. In the current study, we established a circRNA-associated ceRNA network in the PTC. Based on this, six hub PFI-related DE-mRNAs of the PTC were identified. A six-DE-mRNA signature was subsequently established, which was able to distinguish high-risk PTC patients from the low-risk ones and could accurately predict the PFI. Nomogram has been widely applied in oncology to assess the prognosis of cancer patients (Balachandran et al., 2015). Nomogram can integrate various prognostic factors, including molecular and clinical parameters, and provide a visual graphical interface for personalized prediction of clinical events. In this study, to establish a more accurate assessment tool for prognosis evaluation in post-surgical PTC, a prognostic nomogram was established incorporating the DE-mRNA signature and clinicopathological parameters. This nomogram was able to accurately predict PFI of PTC and was better than the ATA risk stratification system, MACIS score, and AJCC staging system recommended by ATA guidelines.

Currently, 11 DE-circRNAs were identified to regulate the six hub DE-mRNAs via the ceRNA mechanism in the study. The role of hsa_circ_0089153 in PTC has previously been reported. hsa_circ_0089153 was upregulated in clinical specimens of PTC (Li et al., 2018). *In vitro* experiments indicated that downregulation of hsa_circ_0089153 expression could inhibit proliferation, migration, and invasion of PTC cells. The luciferase reporter assay confirmed that hsa_circ_0089153 sponged hsa-miR-145 to mediate upregulation of *ZEB2* expression, thereby playing a carcinogenic role in PTC. hsa_circ_0089153 was also reported to be upregulated in gastric cancer and bladder cancer (Wei et al., 2019; Zhuang et al., 2020). Our study indicated that upregulated hsa_circ_0089153 may sponge hsa-miR-139-3p and upregulated *SPC24* and *CDKN2A* expression to promote PTC progression. hsa_circ_0003645 was previously identified to be upregulated in non–small cell lung cancer tissue (Zhang et al., 2018). hsa_circ_0038718 was reported to be upregulated in hepatocellular carcinoma

(Qiu et al., 2019). Our result suggested that hsa_circ_0003645 and hsa_circ_0038718 may also play an oncogenic role as a sponge of hsa-miR-139-3p. The function of hsa_circ_0001917 in PTC has not been reported. However, hsa_circ_0001917 was also identified to be upregulated in hepatocellular carcinoma (Qiu et al., 2019). Our result suggested that the upregulated hsa_circ_0001917 may sponge hsa-miR-139-5p, promoting PTC via upregulation of *RIMS2*. The function of hsa_circ_0005699 in cancer was controversial. hsa_circ_0005699 was previously reported to downregulate *MCM8* and *NCAPD2* expression by acting as a sponge of hsa-miR-504, functioning as tumor suppressor in gastric cancer (Guan et al., 2019). Our study suggested that hsa_circ_0005699 may promote PTC as a sponge of hsa-miR-139-3p and upregulate SPC24 and CDKN2A expression. The function of the remaining six circRNAs in cancer is still known. Our study suggests that the upregulation of hsa_circ_0007146 may promote PTC through the sponging of hsa-miR-139-3p. hsa_circ_0001658, hsa_circ_0008784, and hsa_circ_0000965 may upregulate *RIMS2* expression as a sponge of hsa-miR-139-5p, playing an oncogenic role in PTC. Moreover, downregulated hsa_circ_0008354 and hsa_circ_0049271 may sponge hsa-miR-146b-3p and suppress the expression of *CLCNKB, FBXO27*, and *FXYD6*. The function of these circRNAs in PTC requires further experimental validation.

Six hub DE-mRNAs markedly associated with the PFI of PTC were identified in the current study. Upregulated *SPC24* and *CDKN2A* were identified as targets of hsa-miR-139-3p. The tumor-suppressing role of hsa-miR-139-3p has been identified in multiple tumors (Sannigrahi et al., 2017). *SPC24* is an important component of kinetochore-associated NDC80 complex. This complex mediates chromosome segregation and spindle checkpoint activity. *SPC24* plays an important role in maintaining the integrity of kinetochore (McCleland et al., 2004). *SPC24* was previously identified to be highly expressed in anaplastic thyroid cancer. Knockdown of *SPC24* expression inhibited cell growth and invasion and promoted tumor cell apoptosis (Yin et al., 2017). *SPC24* was also reported to be highly expressed in hepatocellular carcinoma and was an independent predictor of survival (Zhu et al., 2015). Downregulation of *SPC24* inhibited growth and invasion of tumor cells and promoted apoptosis. The oncogenic role of *SPC24* was also identified in breast cancer and lung cancer (Zhou et al., 2017, 2018). Here, *SPC24* was identified to be negatively regulated by hsa-miR-139-3p. This regulatory role between *SPC24* and hsa-miR-139-3p was previously observed in bladder cancer (Yonemori et al., 2016). *CDKN2A* is traditionally known as a tumor-suppressor gene coding for two proteins, including the p16INK4a and p14arf (Bockstaele et al., 2006). *CDKN2A* is involved in cell cycle regulation. However, mounting data suggest that *CDKN2A* may play a dual role in multiple tumors. The p14arf that it codes plays an important role in invasion and metastasis and is associated with a poor prognosis (Fontana et al., 2019). Upregulation of p14arf has been identified in multiple hematological malignancies, aggressive types of B-cell lymphomas, and bladder cancers (Sánchez-Aguilera et al., 2002; Berggren et al., 2003; Lee et al., 2003). The oncogenic function of p14arf is associated with the autophagy regulation (Fontana et al., 2019). Downregulation of p14arf was shown to inhibit progression of lymphomas with the *MYC* mutation (Humbey et al., 2008). Particularly in PTC, p16INK4a, and p14arf coded by *CDKN2A* were both identified to be upregulated in thyroid tumorigenesis (Ferru et al., 2006). Wild-type p14arf has been observed to delocalize into the cytoplasm in aggressive PTC. Here, we further identified that *CDKN2A* was upregulated in PTC and was associated with shorter PFI, and hsa-miR-139-3p was the potential negative regulator of *CDKN2A*. The function of *CDKN2A* in PTC progression requires further study. *RIMS2* was also identified to be upregulated in PTC and was potentially regulated by hsa-miR-139-5p. hsa-miR-139-5p was identified to be downregulated in the primary tumor and further in PTC metastasis (Montero-Conde et al., 2020). hsa-miR-139-5p was also able to be sponged by circBACH2 and relieved the suppression of the target gene *LMO4* in PTC (Cai et al., 2019). *RIMS2* was identified as a novel target of hsa-miR-139-5p in this study. *RIMS2* is a Rab effector and scaffold protein associated with exocytosis (Yoo et al., 2013). It was recently reported to have a high mutation rate in melanoma and mantle cell lymphoma (Hill et al., 2020; Zhang and Xia, 2020). In this study, *RIMS2* was identified as a hub DE-mRNA in the ceRNA network and was associated with PFI of PTC. The function of *RIMS2* in PTC requires further experimental validation.

In this study, downregulated *CLCNKB, FBXO27*, and *FXYD6* were identified to be novel targets of hsa-miR-146b-3p in PTC. hsa-miR-146b-3p was previously reported to be upregulated in PTC and positively associated with central lymph node metastases (Han et al., 2016). hsa-miR-146b-3p may promote invasion and metastasis of PTC by targeting *NF2* (Yu et al., 2018). hsa-miR-146b-3p also targeted *PAX8* in modulating the differentiated phenotype of PTC (Riesco-Eizaguirre et al., 2015). *FXYD6* is known as a specific modulator of Na and K-ATPase and is expressed in multiple epithelial cells of the inner ear (Delprat et al., 2007). In accordance with the current study, *FXYD6* was previously identified to be downregulated in PTC and was associated with poor prognosis (Wu et al., 2019). *CLCNKB* is a voltage-gated chloride channel participating in the regulation of cell volume, membrane potential stabilization, signal transduction, and transepithelial transport (Estévez et al., 2001). *CLCNKB* was previously reported to be downregulated in renal carcinoma (Murakami et al., 2007). Hypermethylation in deletions of *CLCNKB* in renal carcinoma further indicated its tumor-suppressing role in cancer (Girgis et al., 2012). *FBXO27* is a component for substrate recognition in the SCF-type E3 ubiquitin ligase complex (Glenn et al., 2008). Its role in cancer is currently unknown. In this study, *CLCNKB, FBXO27*, and *FXYD6* were identified to be negatively regulated by hsa-miR-146b-3p. Together, hsa_circ_0008354 and hsa_circ_0049271 formed an important part of the PFI-related ceRNA subnetwork we established. The potential tumor-suppressing role of *CLCNKB, FBXO27*, and *FXYD6* in PTC requires further validation.

To the best of our knowledge, the ceRNA network we established and the prognostic DE-mRNA signature we proposed have not been reported previously. The nomogram incorporating the DE-mRNA signature and clinical parameters was robust in predicting PFI of PTC. DE-circRNAs, DE-miRNAs, and DE-mRNAs were potential therapeutic targets for prevention and treatment of recurrent PTC. We acknowledge that our study inevitably has some limitations. First, sequencing data and follow-up data of PTCs in our study were based on TCGA-THCA dataset. Most patients were from North America. Therefore, caution should be exercised in extrapolating the conclusions from findings to other populations. Second, the targeting relationship of ceRNA is based on bioinformatics speculation and requires further experimental validation. Finally, the biological function of certain DE-circRNAs, DE-miRNAs, and DE-mRNAs requires investigation with experiments in PTC.

## Conclusions

This study revealed a circRNA-associated ceRNA network in PTC. Based on survival analysis, a ceRNA subnetwork associated with PFI in PTC was identified. The molecules within the PFI-related subnetwork may represent a promising target for the treatment of patients with recurrent PTC. With the hub DE-mRNAs identified within the subnetwork, a prognostic six-DE-mRNA signature was established. The nomogram incorporating the DE-mRNA signature and clinical parameters is robust in predicting the PFI of post-surgical PTC.

## Data Availability Statement

Publicly available datasets were analyzed in this study. This data can be found here: the datasets analyzed during the current study are available in the Gene Expression Omnibus (https://www.ncbi.nlm.nih.gov/geo/) and the Cancer Genome Atlas (https://portal.gdc.cancer.gov/).

## Author Contributions

ZL, XL, and XX: conception and design. MW, SL, JH, and RL: development of methodology. MW and HY: analysis and interpretation of data. MW: writing of the manuscript. XX, SL, JH, and XL: review of the manuscript. ZL: study supervision. All authors contributed to the article and approved the submitted version.

## Conflict of Interest

The authors declare that the research was conducted in the absence of any commercial or financial relationships that could be construed as a potential conflict of interest. The reviewer CL declared a shared affiliation with the authors to the handling editor at time of review.
