# Supplementary material for: Progression Risk Assessment of Post-surgical Papillary Thyroid Carcinoma Based on Circular RNA-Associated Competing Endogenous RNA Mechanisms
Source: Front Cell Dev Biol. 2021 Jan 21;8:606327. doi: 10.3389/fcell.2020.606327 (PMC7859334; doi:10.3389/fcell.2020.606327)
Supplement: Supplementary file 1 [file Data_Sheet_1.ZIP › Supplementary materials/Supplementary_Material.docx]

Supplementary Material

# Supplementary Figures


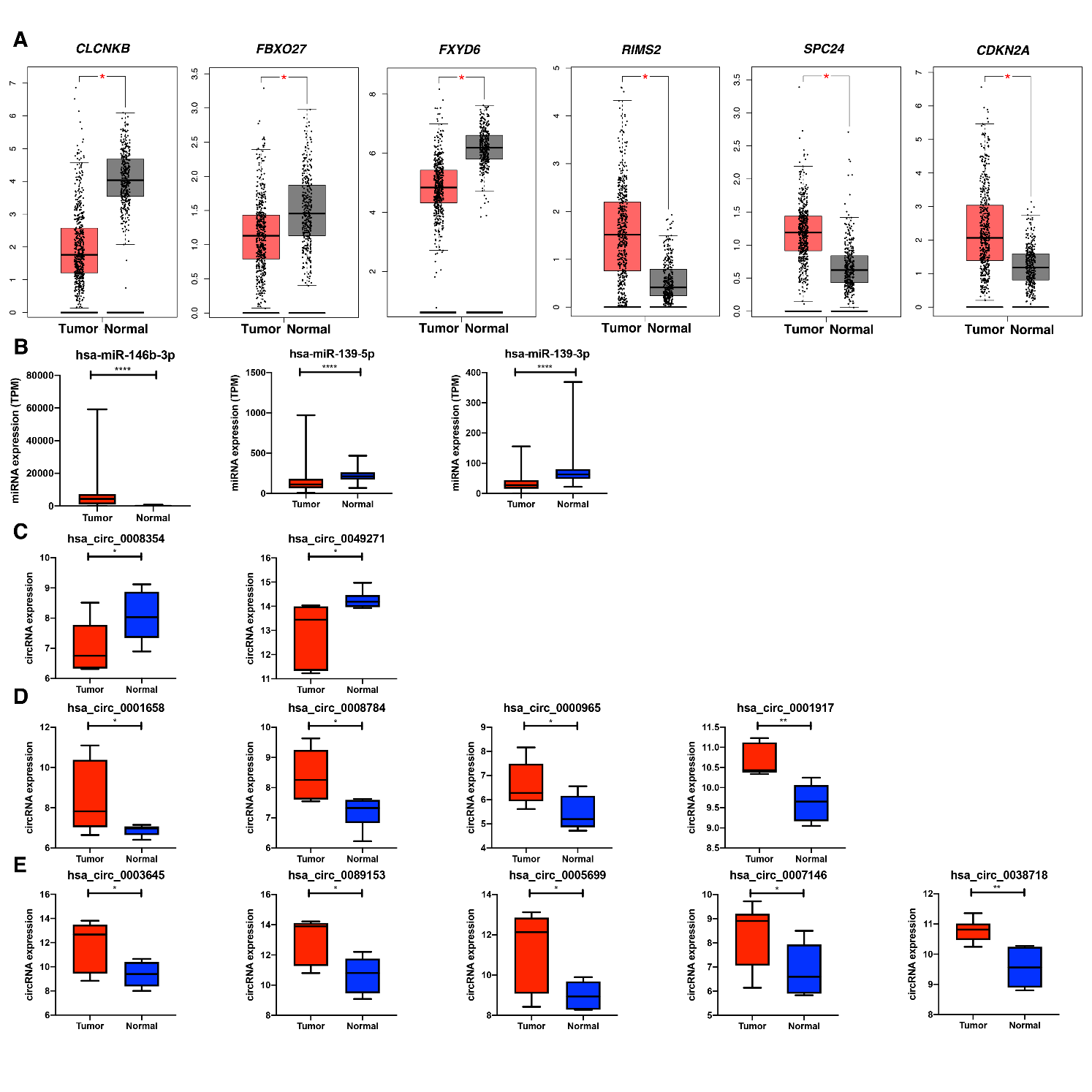


**Figure S1** (A) shows validation of the differential expression levels of the hub DE-mRNAs (*CLCNKB, FBXO27, FXYD6, RIMS2, SPC24* and *CDKN2A*) in the PTC tumor tissues and the normal tissues using GEPIA (http://gepia.cancer-pku.cn). (B) shows the differential expression of the three DE-miRNAs associated with the hub DE-mRNAs in TCGA-THCA dataset. *CLCNKB, FBXO27* and *FXYD6* are negatively regulated by hsa-miR-146b-3p. *RIMS2* is negatively regulated by hsa-miR-139-5p. *SPC24* and *CDKN2A* are negatively regulated by hsa-miR-139-3p. (C)shows the differential expression of hsa_circ_0008354 and hsa_circ_0049271 in GSE93522. These DE-circRNAs may sponge hsa-miR-146b-3p within the ceRNA network. (D) shows the differential expression of hsa_circ_0001658, hsa_circ_0008784, hsa_circ_0000965 and hsa_circ_0001917 in GSE93522. These DE-circRNAs may sponge hsa-miR-139-5p within the ceRNA network. (E) shows the differential expression of hsa_circ_0003645, hsa_circ_0089153, hsa_circ_0005699, hsa_circ_0007146 and hsa_circ_0038718 in GSE93522. These DE-circRNAs may sponge hsa-miR-139-3p within the ceRNA network. *P < 0.05, **P < 0.01. *** P < 0.001. **** P < 0.0001.

# Supplementary Tables

**Table S1.** Differentially expressed circRNAs identified in PTC

**Table S2.** Differentially expressed miRNAs identified in PTC

**Table S3.** Differentially expressed mRNAs identified in PTC

**Table S4.** The binding miRNAs of circRNAs predicted by Circular RNA Interactome

**Table S5.** The target mRNAs of differentially expressed miRNAs predicted by miRWalk 3.0

**Table S6.** Gene Enrichment Analysis

**Table S7.** Correlation analysis of miRNAs and potential target genes in TCGA-THCA dataset

**Table S8.** Gene Set Enrichment Analysis

**Table S9.** Reasons for exclusion from the evaluation of prognostic factors and establishment of nomogram
