# Supplementary figures and images for: Progression Risk Assessment of Post-surgical Papillary Thyroid Carcinoma Based on Circular RNA-Associated Competing Endogenous RNA Mechanisms
Source: Front Cell Dev Biol. 2021 Jan 21;8:606327. doi: 10.3389/fcell.2020.606327 (PMC7859334; doi:10.3389/fcell.2020.606327)

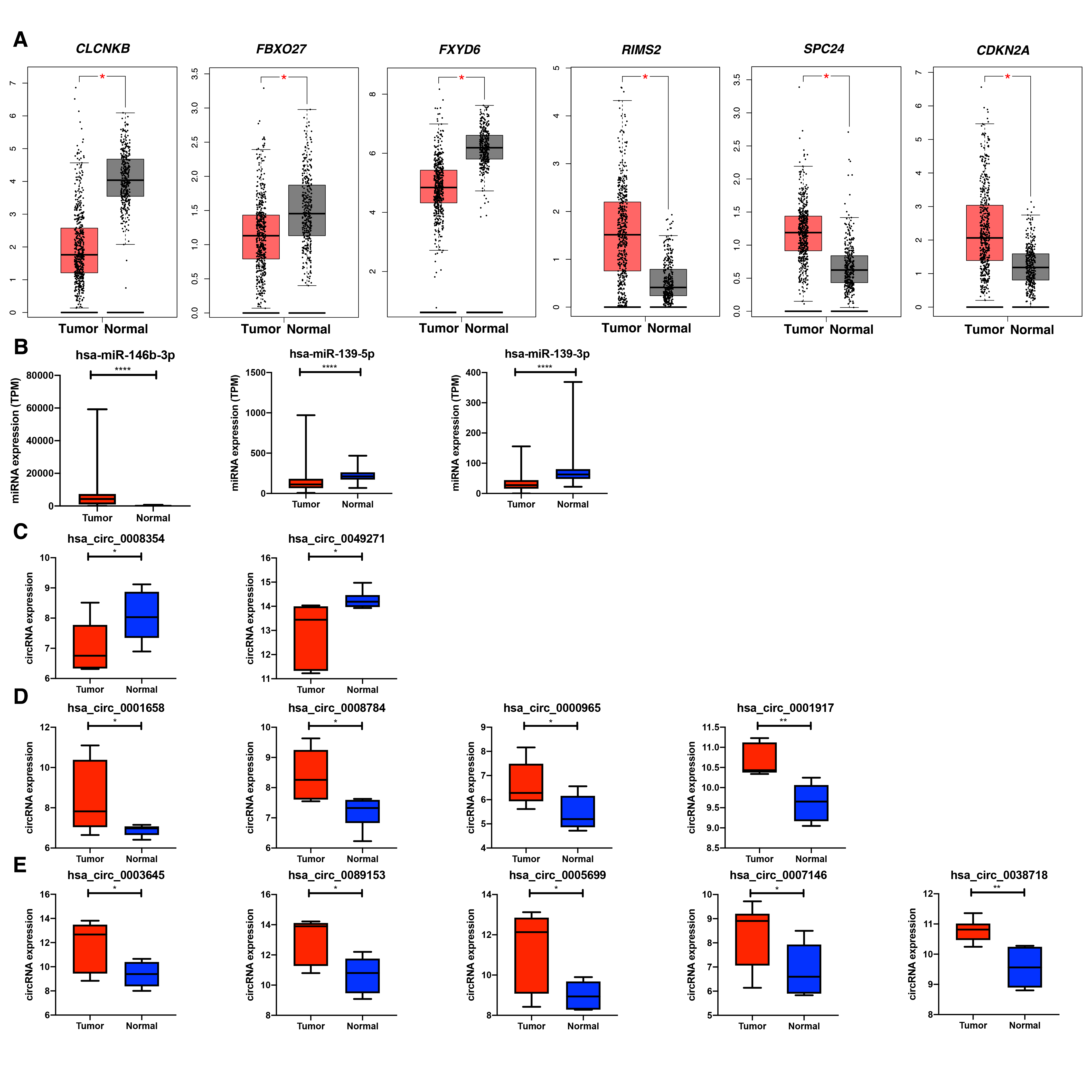

Supplement: Supplementary file 1 [file Data_Sheet_1.ZIP › Supplementary materials/Figure S1.tif]
